# Stem cell transplantation uncovers TDO-AHR regulation of lung dendritic cells in herpesvirus-induced pathology

**DOI:** 10.1172/jci.insight.139965

**Published:** 2021-01-25

**Authors:** Stephen J. Gurczynski, Nicolas L. Pereira, Steven M. Hrycaj, Carol Wilke, Rachel L. Zemans, Bethany B. Moore

**Affiliations:** 1 Division of Pulmonary & Critical Care Medicine, Department of Internal Medicine, and; 2 Department of Microbiology and Immunology, University of Michigan, Ann Arbor, Michigan, USA.

**Keywords:** Immunology, Transplantation, Cytokines, Dendritic cells, Fibrosis

## Abstract

The aryl-hydrocarbon receptor (AHR) is an intracellular sensor of aromatic hydrocarbons that sits at the top of various immunomodulatory pathways. Here, we present evidence that AHR plays a role in controlling IL-17 responses and the development of pulmonary fibrosis in response to respiratory pathogens following bone marrow transplant (BMT). Mice infected intranasally with gamma-herpesvirus 68 (γHV-68) following BMT displayed elevated levels of the AHR ligand, kynurenine (kyn), in comparison with control mice. Inhibition or genetic ablation of AHR signaling resulted in a significant decrease in IL-17 expression as well as a reduction in lung pathology. Lung CD103^+^ DCs expressed AHR following BMT, and treatment of induced CD103^+^ DCs with kyn resulted in altered cytokine production in response to γHV-68. Interestingly, mice deficient in the kyn-producing enzyme indolamine 2-3 dioxygenase showed no differences in cytokine responses to γHV-68 following BMT; however, isolated pulmonary fibroblasts infected with γHV-68 expressed the kyn-producing enzyme tryptophan dioxygenase (TDO2). Our data indicate that alterations in the production of AHR ligands in response to respiratory pathogens following BMT results in a pro-Th17 phenotype that drives lung pathology. We have further identified the TDO2/AHR axis as a potentially novel form of intercellular communication between fibroblasts and DCs that shapes immune responses to respiratory pathogens.

## Graphical abstract

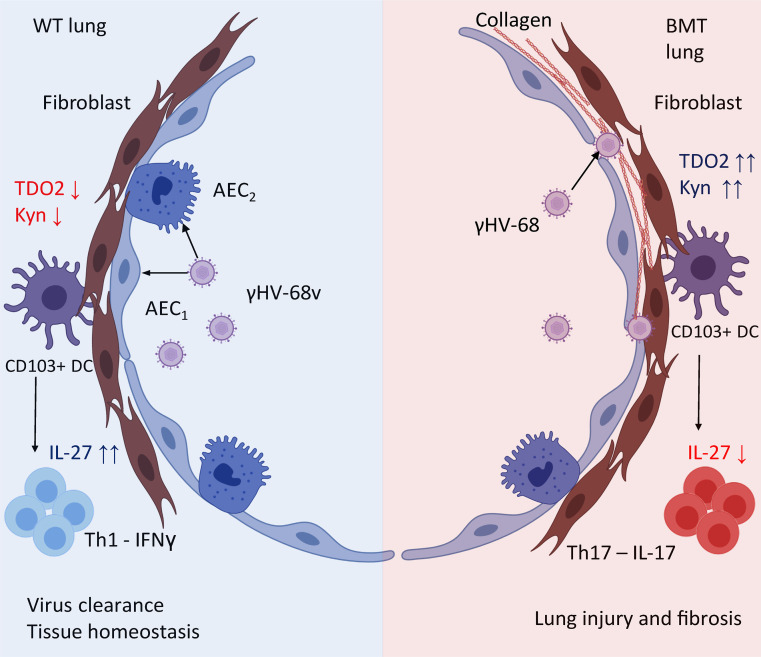

## Introduction

Bone marrow transplantation (BMT) is a curative therapy for a variety of malignant and autoimmune diseases; however, many patients who undergo BMT will develop severe and often life-threatening pulmonary complications. Because of the immune-compromised state following BMT, reactivation of latent herpesvirus infections is common, and first-onset herpesvirus infections are linked to the development of severe post-BMT pulmonary complications, like idiopathic pneumonia syndrome, which have high associated morbidity and mortality rates (1). We have previously published that BMT mice infected with the murine-specific gamma-herpesvirus, γHV-68, develop a severe pneumonitis and pulmonary fibrosis by 21 days postinfection (dpi), which mimics many of the clinical manifestations of the pulmonary complications seen after BMT (2). It is clear that alterations to immune function following BMT are widespread and involve myriad leukocyte subsets, such as lymphocytes, macrophages, and dendritic cells (DCs) (3–5). IL-17 also plays a central role in post-BMT pathology both in humans and in mouse models (2, 6). The upstream pathways that promote IL-17 over other protective cytokines following BMT, however, remain understudied.

The aryl-hydrocarbon receptor (AHR) is an intracytoplasmic receptor that senses a variety of polyaromatic hydrocarbons. The immune-modulating capabilities of the AHR have long been appreciated since its original description as the main receptor for the xenobiotic, and potent carcinogen, dioxin. Since this initial characterization, a variety of endogenous AHR ligands have been described; many of these ligands are metabolites of various aromatic amino acids, such as tryptophan (7). The most well studied of these endogenous ligands belong to the kynurenine (kyn) family of tryptophan metabolites. Kyn is produced through a cascade of enzymatic steps known collectively as the kynurenine pathway (KP) (8). In the first rate-limiting step of the KP, tryptophan is oxidized by several cellular heme-dependent oxygenases, most notably, indolamine 2-3 dioxygenase (IDO1) and tryptophan dioxygenase (TDO2). IDO and TDO2 and their role in producing immune-modulating kyn have been studied extensively in the field of cancer immunobiology, but these enzymes are less well studied regarding their potential role during the pathogenesis of organ fibrosis (9). Notably, the IDO1/AHR axis was recently shown to drive, at least in part, severe lung inflammation and pulmonary fibrosis following allogeneic stem cell transplant (10). However, IDO1 deficiency attenuated CCL4-induced liver fibrosis by a mechanism involving a compensatory upregulation of TDO2 and overproduction of kyn (11). Both of these mechanisms involved modulation of Th17 cell differentiation, highlighting the central role of AHR in modulating production of proinflammatory cytokines, especially IL-17. However, to our knowledge, the role of the other major kyn-producing enzyme, TDO2, has not been studied in the context of pulmonary inflammation or lung fibrosis, nor has it been shown to play a pathogenic role in the presence of the more ubiquitous IDO1 enzyme.

AHR signaling is complex; however, the most well-studied signaling cascade involves AHR in a non–ligand-bound form complexed with various heat shock/chaperone proteins, including HSP90, XAP2, and P23 (12, 13). Dubbed canonical AHR signaling, upon ligand binding, AHR transits to the nucleus, where it disassociates from the chaperone protein scaffold and binds with the aryl-hydrocarbon nuclear translocator (ARNT), a member of the hypoxia-inducible factor family of transactivators (14). Dimerized AHR-ARNT then binds genomic xenobiotic response elements, which facilitate transcription of a wide variety of genes, many of which, such as the *Cyp* genes, are members of the cytochrome p450 family of enzymes, which aid in detoxifying cells of the many xenobiotics that AHR binds (15).

More recently, other AHR dimerizing partners have been described in what is known as noncanonical AHR signaling. Noncanonical signaling of AHR is increased under inflammatory conditions and shifts AHR binding to proinflammatory transactivators, such as the p65 subunit of the inflammatory transcription factor NF-κB (RelA) (16–18). AHR complexed, or in association with NF-κB, can modulate inflammatory responses, and while it is still unclear what specific factors mediate the induction of canonical versus noncanonical AHR signaling, Toll-like receptor (TLR) stimulation is thought to play a central role and is especially important in AHR signaling in leukocytes such as DCs, macrophages, and T cells (17, 19).

Here we present data that AHR signaling in response to gamma-herpesviruses is increased following BMT. Specifically, activation of AHR signaling on CD103^+^ DCs diminished their capacity to inhibit Th17 differentiation via reduction in Th17-suppressive cytokines, such as IL-27. We further show that production of the AHR ligand kyn is elevated following BMT and that herpesvirus infection drives expression of the kyn-producing enzyme, TDO2, in infected fibroblasts but not epithelial cells. This work sheds mechanistic light on how cellular crosstalk between nonimmune cells, e.g., fibroblasts, and immune cells like DCs occurs during complex pathologies and brings to light the possibility of utilizing AHR modulation as an immunotherapy for post-BMT pulmonary pathologies and pulmonary fibrosis. This work also provides potentially novel evidence for a specific role for TDO2 in mediating lung pathology even in the presence of widely expressed IDO1.

## Results

### Ablation of AHR signaling rescues γHV-68–induced fibrosis following BMT.

**Figure 1:**
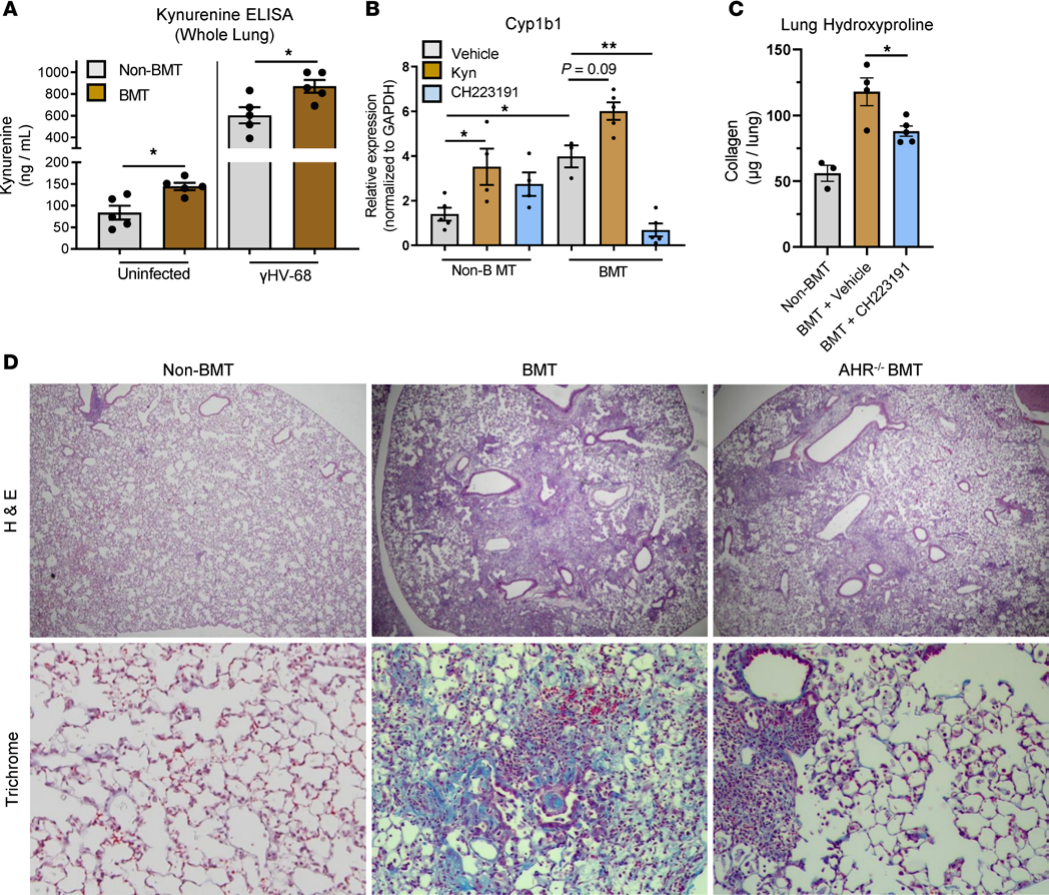
Ablation of AHR signaling rescues γHV-68–induced fibrosis following BMT. (**A**) Concentration of kyn was assessed in whole-lung homogenates by ELISA from groups of BMT (mean ± SEM, *n* = 5) or non-BMT (mean ± SEM, *n* = 5) mice infected with γHV-68 or mock infected for 7 days. (**B**) Relative quantity of Cyp1b1 transcript was determined from lung leukocytes isolated from collagenase-digested lungs of either non-BMT (shown as mean ± SEM, vehicle treated *n* = 5, kyn treated *n* = 4, CH223191 treated *n* = 4) or BMT (shown as mean ± SEM, vehicle treated *n* = 3, kyn treated *n* = 5, CH223191 treated *n* = 5) mice infected with γHV-68 for 7 days. (**C**) Groups of non-BMT or BMT mice were infected with γHV-68 and treated with the AHR inhibitor, CH223191, 10 μg, every 2 days for 14 days. At 21 dpi lungs were harvested, and collagen content was assessed by hydroxyproline assay (shown as mean ± SEM, non-BMT *n* = 5, BMT + vehicle *n* = 4, BMT + CH223191 *n* = 5). (**D**) Sections from formalin-fixed paraffin-embedded mouse lung were prepared and stained with H&E or trichrome to detect collagen. Statistical significance was determined by ANOVA, *P < 0.05, **P < 0.01.

Activation of the AHR pathway via promotion of the KP is well studied in various inflammatory contexts (20–22). However, little is known about activation of the KP and AHR in the setting of viral infections following BMT. Thus, we utilized a model of gamma-herpesvirus infection following BMT using a C57BL/6J syngeneic BMT model and i.n. infection with the murine gamma-herpesvirus, γHV-68. After 5 weeks of BMT reconstitution, we analyzed lung-associated concentrations of the main KP metabolite, kyn, following 7 days of herpesvirus infection compared with noninfected mice (Figure 1A). Although kyn concentration was increased even in non-BMT mice infected with γHV-68, there was an additional increase in lung-associated kyn in γHV-68 BMT mice when compared with non-BMT mice (Figure 1A). Kyn is a well-characterized endogenous ligand for AHR, and thus we next measured AHR activation by looking at expression of the AHR-specific target transcript, *Cyp1b1*, in leukocytes isolated from collagenase-digested lungs. In agreement with the concentrations of kyn, we observed an increase in the expression of *Cyp1b1* expression in BMT mice, compared with non-BMT mice, following γHV-68 infection (Figure 1B, gray bars). To determine if further modulation of AHR signaling could alter Cyp1b1 expression, we treated leukocytes with either purified kyn or the specific AHR antagonist CH223191. We observed a large increase in Cyp1b1 expression in BMT leukocytes treated with kyn (tan bars), which could be inhibited by treatment with AHR antagonist (Figure 1B, blue bars). Further, expression of *Cyp1b1* was as high at baseline in BMT leukocytes as WT leukocytes treated with exogenous kyn. Together, these data indicate that both the KP and AHR pathways are augmented in response to BMT and that they are further augmented following viral infection.

We next determined if modulation of AHR signaling could alter pathological outcomes in γHV-68–infected mice following BMT. To this end, we treated groups of BMT or control mice, infected with γHV-68, every 2 days with the AHR antagonist CH223191 beginning at 1 dpi. At 21 dpi, lungs were harvested, and collagen content, a surrogate marker for fibrosis, was measured by hydroxyproline assay. In agreement with previously published work, BMT mice exhibited significant accumulation of collagen by 21 dpi (Figure 1C). Treatment with CH223191 was able to significantly reduce collagen content, indicating that inhibition of AHR signaling can rescue fibrotic pathology in BMT mice. To further confirm the role of AHR in the development of pneumonitis and fibrosis following BMT, mice were transplanted with AHR^–/–^ bone marrow and infected with γHV-68. At 21 dpi, lungs were harvested, and histopathology was analyzed from sectioned lung tissue. AHR^–/–^ BMT mice exhibited a decrease in overall inflammatory infiltration and a decrease in fibrotic pathology as evidenced by H&E and trichrome straining, respectively (Figure 1D). Together these data indicate a role for AHR signaling in the development of virus-induced post-BMT lung pathology.

### Ablation of AHR signaling decreases the Th17/IL-17 burden in γHV-68–infected BMT mice.

**Figure 2:**
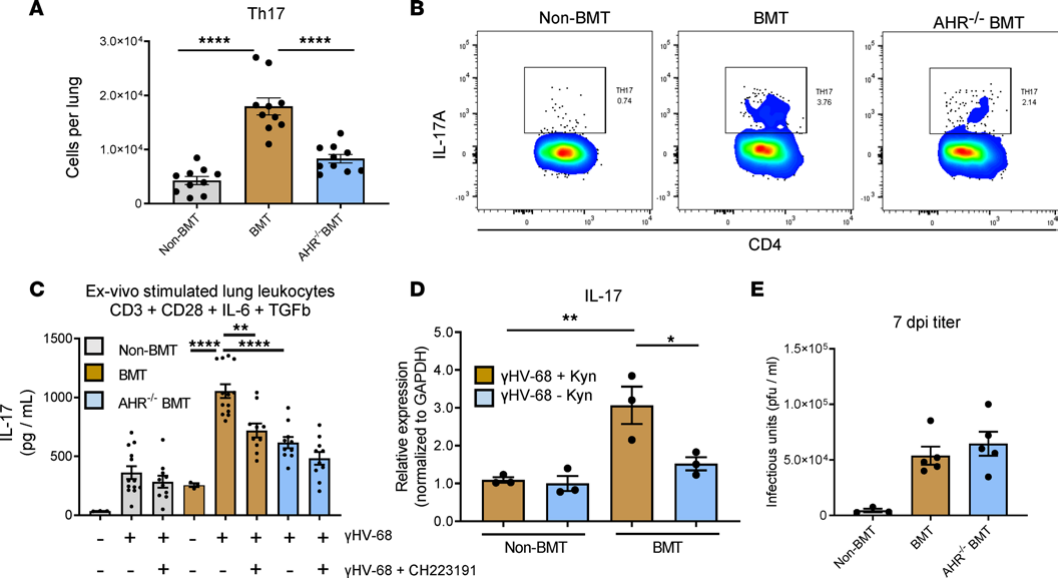
Ablation of AHR signaling decreases the Th17/IL-17 burden in γHV-68–infected BMT mice. (**A**) Groups of non-BMT, BMT, or AHR^–/–^ BMT mice (shown as mean ± SEM, *n* = 10 mice per group) were infected with γHV-68 for 7 days. Lungs were collagenase digested and isolated leukocytes were stimulated with PMA and ionomycin. Numbers of IL-17^+^CD3^+^CD4^+^Thy1.2^+^ lymphocytes were determined by flow cytometry. (**B**) Representative plots from data in **A**. (**C**) Lung leukocytes were isolated from groups of non-BMT, BMT, or AHR^–/–^ BMT mice by collagenase digestion (*n* = 8–13 mice per group). Cells were treated with either vehicle control (shown as mean ± SEM, *n* = 3), γHV-68 + vehicle (non-BMT *n* = 13, BMT *n* = 13, AHR^–/–^ *n* = 10), or a combination of 200 μM kyn and 10 μM CH223191 and γHV-68 (non-BMT *n* = 10, BMT *n* = 10, AHR^–/–^ *n* = 10) for 24 hours. Concentration of IL-17 was measured in cell culture supernatants by ELISA. (**D**) Lung leukocytes were isolated from either non-BMT (shown as mean ± SEM, *n* = 3) or BMT (shown as mean ± SEM, *n* = 3) mice and stimulated with 200 μM kyn for 24 hours. Transcript expression was quantified by quantitative reverse transcription PCR (qRT-PCR). (**E**) Groups of non-BMT (shown as mean ± SEM, *n* = 5), BMT (shown as mean ± SEM, *n* = 5), or AHR^–/–^ BMT mice (shown as mean ± SEM, *n* = 5) were infected with γHV-68. At 7 dpi, infectious virus was measured from whole-lung homogenate via plaque assay. Statistical significance was determined by ANOVA, *P < 0.05, **P < 0.01, ****P < 0.0001.

We have previously published that the pneumonitis and fibrosis seen in BMT mice in response to viral infection are IL-17A dependent (2). Given that AHR has previously been shown to control expression of IL-17A and that inhibition of AHR signaling rescued post-BMT pathology, we asked whether AHR signaling contributes to the expression of IL-17 following BMT. To address this, we again utilized AHR^–/–^ BMT mice and analyzed numbers of Th17 cells (Thy1.2^+^CD3^+^CD4^+^IL-17*^+^*) by flow cytometry. At 7 dpi, AHR^–/–^ mice exhibited a 2-fold decrease in the number of Th17 cells in comparison with WT-BMT mice (Figure 2A, representative flow plots shown in Figure 2B). To further address the contribution of AHR signaling to the production of IL-17, lung-associated leukocytes were purified by collagenase digestion and stimulated with the AHR inhibitor CH223191 in the presence of the IL-17–stimulating cytokines IL-6 and TGF-β. Following 48 hours of stimulation, BMT leukocytes produced significantly more IL-17 in comparison with WT leukocytes (Figure 2C). Inhibition of AHR signaling significantly decreased production of IL-17 from BMT leukocytes but had little effect on non-BMT leukocytes. Leukocytes isolated from AHR^–/–^ BMT mice produced significantly less IL-17 than WT-BMT leukocytes and were nonresponsive to inhibition by CH223191 (Figure 2C). Expression of IL-17 transcript was significantly elevated in BMT leukocytes in response to kyn treatment (Figure 2D). Further, inhibition of AHR signaling did not influence production of infectious virus as measured by plaque assay at 7 dpi (Figure 2E), indicating that AHR contributes to cytokine secretion and plays a role in progression of post-BMT fibrosis but does not influence herpesvirus replication within the lung overall.

### Distribution of the AHR on myeloid cells is altered following BMT.

**Figure 3:**
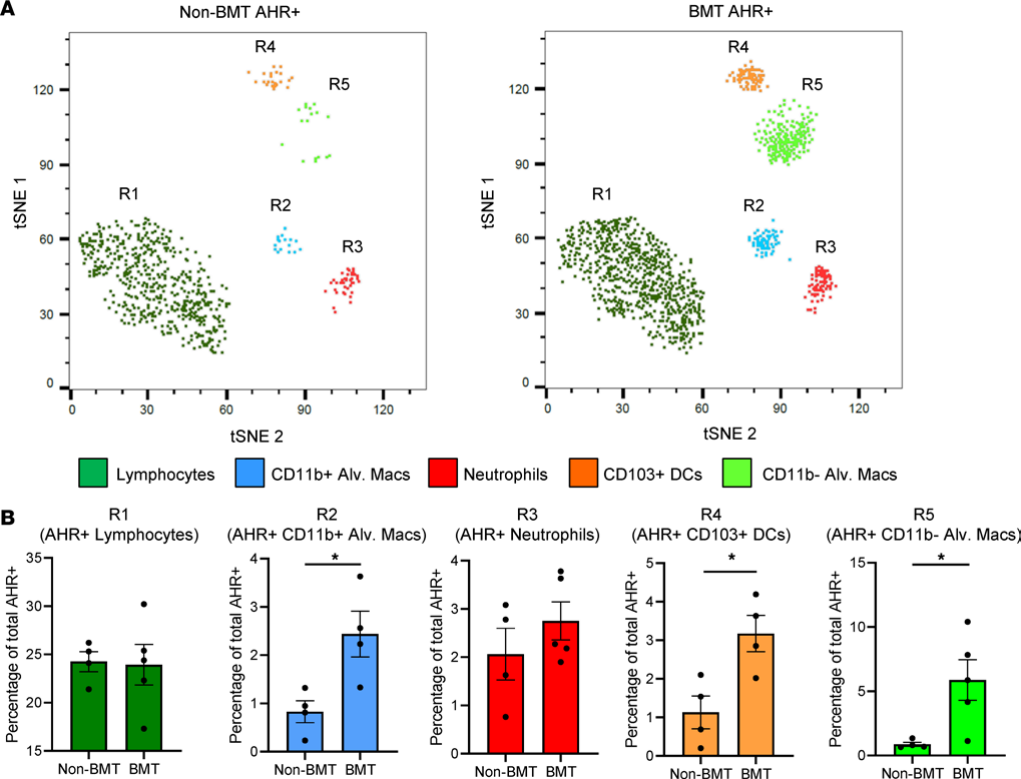
Distribution of the AHR on myeloid cells is altered following BMT. (**A**) Groups of non-BMT (shown as mean ± SEM, *n* = 4) or BMT (shown as mean ± SEM, *n* = 5) mice were infected with γHV-68 for 7 days. Lungs were collagenase digested and leukocytes were analyzed for expression of AHR by flow cytometry using the following markers: R1 lymphocytes (CD45^+^CD11b/c^–^Thy1.2^+^). R2 CD11b^+^ alv. macs (CD45^+^CD11b^+^CD11c^–^CD24^+^CCR3^+^SiglecF^+^). R3 neutrophils (CD45CD11b^+^Ly6g^+^). R4 CD103^+^ DCs (CD45^+^CD11c^+^CD11b^–^CD24^+^CD103^+^). R5 CD11b^–^ alv. macs (CD45^+^CD11c^+^CD11b^–^CD64^+^SiglecF^+^). tSNE, t-distributed stochastic neighbor embedding. (**B**) Quantification of cells from **A**. Statistical significance was determined by Student’s *t* test. *P < 0.05.

AHR has a broad cellular distribution and is present in lymphocytes, dendritic cells, macrophages, and epithelial cells. To determine if the distribution of AHR was altered following BMT, we utilized multicolor flow cytometry in combination with multidimensional data reduction analysis to identify the leukocyte populations that expressed AHR following γHV-68 infection. Overall, there was an increase in the total number of CD45^+^ cells that expressed AHR following BMT (Figure 3A). Multidimensional data reduction (tSNE) revealed populations of AHR^+^CD103^+^ DCs and alveolar macrophages, which increased in BMT mice in comparison with WT (Figure 3B and Supplemental Figure 1; supplemental material available online with this article; https://doi.org/10.1172/jci.insight.139965DS1). However, no significant differences were observed in AHR^+^ lymphocytes and neutrophils compared to WT mice (Figure 3B).

### Altered cytokine production from CD103^+^ DCs in the presence of the AHR agonist, kyn.

**Figure 4:**
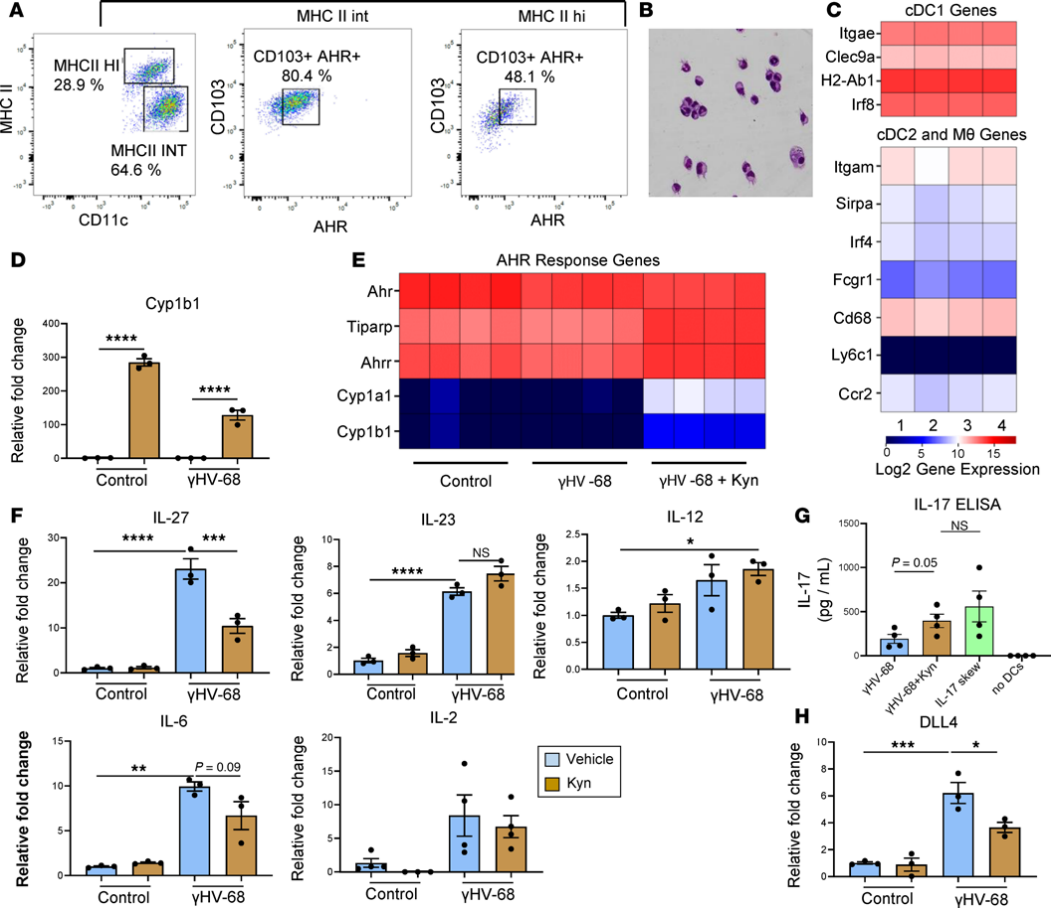
Altered cytokine production from CD103^+^ DCs in the presence of the AHR agonist, kyn. (**A**) Flow plots showing purity of ex vivo–generated iCD103^+^ DCs. Cells were first gated using a live/dead stain and then on CD11c and MHCII following by gating on CD103 and AHR. (**B**) Bright-field micrograph of ex vivo–generated CD103^+^ DCs. Original magnification ×60. (**C**) RNA-Seq analysis of transcripts expressed in 4 separate iCD103 cultures. Genes listed are as follows: cDC1 genes: Itgae (CD103), Clec9a, H2-Ab1 (MHCII), Irf8. cDC2 genes: Itgam(CD11b), Sirpα, Irf4, Fcgr1 (CD64), CD68, Ly6c1 (CCR2). (**D**) Expression of the AHR response gene Cyp1b1 was determined by qRT-PCR following 24 hours of stimulation with kyn and/or γHV-68 (blue bars: vehicle treated, not visible due to scale, *n* = 3; brown bars: kyn treated, *n* = 3, shown as mean ± SEM). (**E**) RNA-Seq analysis of AHR response genes from iCD103 cultures treated with γHV-68, γHV-68 + kyn, or vehicle control (*n* = 4 cultures per group). (**F** and **H**) Ex vivo–generated CD103^+^ DCs were treated with kyn and either infected with γHV-68 or mock infected. Twenty-four hours after treatment total RNA was extracted, and gene expression was quantified by qRT-PCR for the indicated transcript (shown as mean ± SEM, *n* = 3 per group except IL-2, where *n* = 4 per group). (**G**) ICD103^+^ DCs were cocultured with naive CD4^+^ T cells in the presence of 1 μg/mL anti-CD3 and anti-CD28. ICD103^+^ DCs were pretreated overnight with γHV-68 alone (MOI = 1.0) or in combination with 200 μM kyn, DCs were washed once with complete media before plating at a 10:1 T cell/DC ratio. As a positive control, CD4^+^ T cells were skewed with 20 ng/mL recombinant IL-6 and 2 ng/mL recombinant TGF-β with anti-CD3 and anti-CD28 but without DC stimulation (IL-17 skew) or T cells alone without further stimulation (no DCs) shown as mean ± SEM, *n* = 4 per group. Statistical significance was determined by ANOVA, *P < 0.05, **P < 0.01, ***P < 0.001, ****P < 0.0001.

We previously published that CD103^+^ DC function is altered in response to herpesviruses following BMT (2, 4, 5). Given the marked increase in the number of AHR^+^CD103^+^ DCs in the lungs of BMT mice, we next addressed the question of whether AHR signaling could alter CD103^+^ DC function in response to γHV-68. CD103^+^ DCs can be cultured ex vivo from bone marrow stem cells using a combination of recombinant murine GM-CSF and FMS-like tyrosine kinase 3 ligand (Flt3L) and are termed induced CD103^+^ (iCD103^+^) DCs (23). We utilized this approach to generate a population of iCD103^+^ DCs that expressed high levels of CD11c and MHCII and that morphologically looked like DCs (Figure 4, A and B). Importantly, approximately 50% of these ex vivo–generated iCD103^+^ DCs expressed AHR (Figure 4A). Further, RNA-Seq analysis of these iCD103^+^ DCs indicated elevated expression of the conventional type 1 DC (cDC1) markers Itgae (CD103), Clec9a, and Irf8 (Figure 4C) and comparatively low expression levels of the cDC2 markers Itgam (CD11b, Sirpα, and Irf4) or the common macrophage markers CD64, CD68, Ly6c, and CCR2 (Figure 4C), indicating that iCD103^+^ cells differentiated ex vivo in the presence of GM-CSF and Flt3L closely resemble in vivo cDC1 (CD103^+^ DCs) (24, 25). We next treated these iCD103^+^ DCs with γHV-68 in the presence of the AHR ligand kyn or vehicle control for 24 hours. Following kyn treatment there was a large increase in Cyp1b1 expression in both control as well as γHV-68–treated iCD103^+^ DCs as indicated by both qRT-PCR and RNA-Seq analysis (Figure 4, D and E). We further noted increased expression of the AHR response genes TiPARP, AHRr, and Cyp1a1 by RNA-Seq following kyn treatment, indicating that the AHR pathway was activated (Figure 4E).

We next addressed the effects of kyn stimulation on production of common DC cytokines. Infection with γHV-68 alone induced expression of multiple cytokines, including IL-27, IL-23, IL-12, IL-6, and IL-2, while stimulation with kyn alone failed to increase cytokine expression (Figure 4F). When iCD103^+^ DCs were infected with γHV-68 in the presence of kyn, however, a significant reduction in IL-27 expression was noted. We further noted a trend toward lower expression of IL-6 following kyn treatment; however, expression of all the other cytokines tested, i.e., IL-23, IL-12, and IL-2, remained unchanged (Figure 4F).

Because IL-27 has been implicated in the suppression of Th17 differentiation, we next asked whether CD4^+^ T cells coincubated with iCD103^+^ DCs stimulated with kyn would exhibit increased Th17 differentiation in response to γHV-68. Low levels of IL-17 were detected in coculture supernatants in response to γHV-68 alone (Figure 4G). However, CD4^+^ T cells secreted almost 2-fold more IL-17 in the presence of DCs that had been pretreated with kyn and γHV-68, conditions that mimic in vivo infection with γHV-68 in BMT mice (Figure 4G). Interestingly, the level of IL-17 observed from kyn-stimulated DC coculture was similar to T cells that had been skewed under highly polarizing Th17-skewing conditions (Figure 4G). To address whether the effect of AHR activation on APC cytokine secretion was specific to CD103^+^ DCs, we infected either primary alveolar macrophages from the lungs of naive mice or bone marrow–derived macrophages with γHV-68 alone or in combination with kyn. In both cases, expression of the AHR response gene *Cyp1b1* was increased with the addition of kyn; however, no change in expression of IL-27 was observed in either macrophage population, indicating that the effect of AHR on cytokine secretion may be unique to CD103^+^ DCs (Supplemental Figure 2). We previously published that DCs in BMT mice expressed lower levels of the Notch ligand DLL4 in response to herpesvirus infection, ultimately resulting in decreased Th1 skewing and increased Th17 skewing of lymphocytes, which contributed to post-BMT pneumonitis and fibrosis (4). Interestingly, we noted significantly decreased expression of DLL4 in iCD103^+^ DCs treated with both γHV-68 and kyn in comparison with γHV-68 alone (Figure 4H). Together, these data indicate that iCD103^+^ DCs both express AHR and can influence the differentiation of Th17 cells in response to AHR activation.

**Figure 5:**
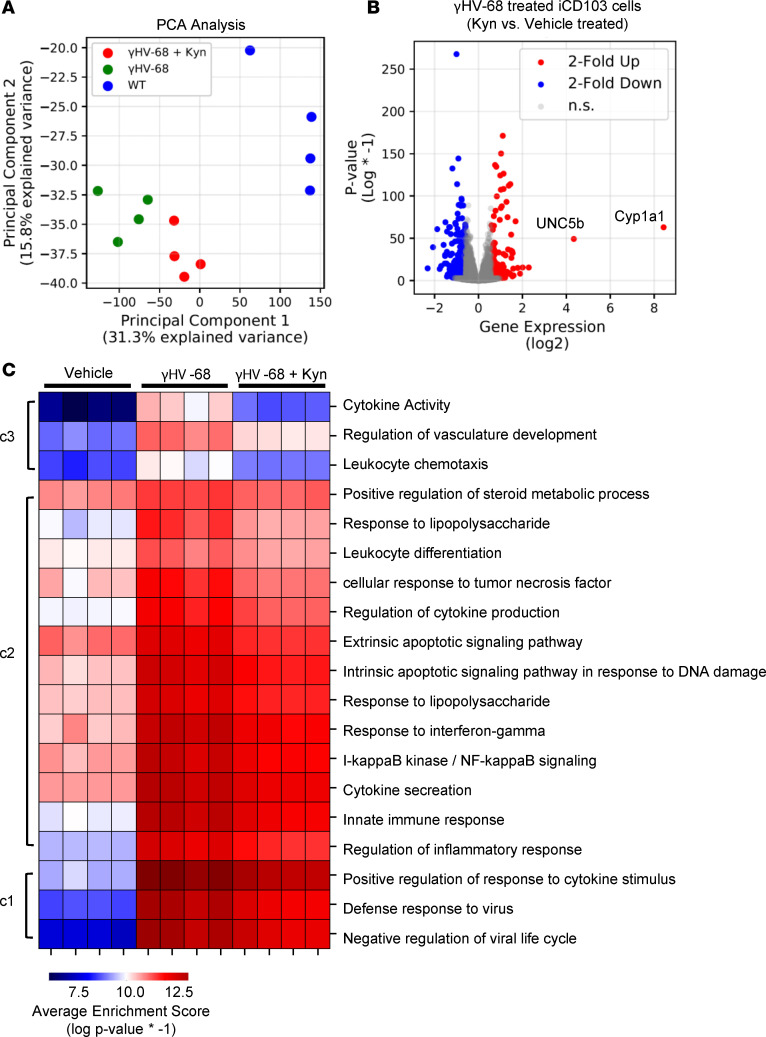
Kyn suppresses the immunological function of iCD103^+^ DCs. ICD103^+^ cells (*n* = 4 per group) were treated with γHV-68, kyn, or a combination of the two and analyzed via RNA-Seq. (**A**) PCA of normalized transcript expression data for all mapped reads. (**B**) Differential expression analysis of γHV-68–treated iCD103^+^ cells treated with kyn versus cells treated with vehicle alone. Red dots represent genes differentially expressed by ≥1.5-fold (*P* < 0.05). Blue dots represent genes differentially expressed by ≤1.5-fold (*P* < 0.05). Notable upregulated genes are marked with text labels. (**C**) Gene Ontology analysis of the DEGs from **B**.

To further characterize the transcriptional changes induced by AHR activation in iCD103^+^ cells, we conducted RNA-Seq analysis of these cells treated with γHV-68 with or without costimulation with kyn. Principal component analysis (PCA) of iCD103^+^ cells showed marked separation between baseline iCD103^+^ cells and those treated with γHV-68 (Figure 5A). A smaller, but still distinct, effect was noted in iCD103^+^ cells treated with both γHV-68 and kyn in comparison with virus alone, indicating that, although large transcriptional changes are induced by herpesvirus stimulation, there was indeed a detectable effect of AHR activation on the transcription profile of γHV-68–treated DCs.

We next conducted differential expression analysis using R, Python, and DESeq2 to identify differentially expressed genes (DEGs) between iCD103^+^ cells treated with γHV-68 alone and cells treated with both γHV-68 and kyn. In total there were 392 DEGs (135 upregulated and 257 downregulated) identified using a 1.5-fold change cutoff with a *P* value less than 0.05 (Figure 5B). Two of the most highly upregulated DEGs identified, Cyp1a1 and UNC5b (335.5-fold and 20.5-fold upregulated, respectively), are known targets of AHR signaling, thus validating that AHR signaling was indeed activated in kyn-treated cells (26). Interestingly, many of the genes identified as being differentially downregulated in kyn-treated iCD103^+^ DCs by RNA-Seq were cytokines identified previously via qRT-PCR, namely, IL-27 and IL-6 as well as DLL4 (2.4-fold, 2.7-fold, and 1.8-fold downregulated, respectively). Additionally, we noted marked downregulation of other proinflammatory cytokines, such as IL-1β, the type I interferon IFN-β, and TNF-α (3.7-fold, 2.6-fold, and 2.4-fold downregulated, respectively). (A full list of DEGs identified between γHV-68–treated and γHV-68 + kyn–treated iCD103^+^ DCs is supplied in Supplemental Table 1.)

To further identify biological themes of AHR stimulation in iCD103, we utilized Cytoscape with the ClueGO plugin to group DEGs identified in Figure 5B based on Gene Ontology Biological Process (GOBP) information. ClueGO initially grouped the 392 DEGs into 132 GOBP categories (*P* < 0.05; a full list of GOBP categories and associated genes is supplied as Supplemental Table 2). Using Python, we were able to parse the initial GOBP table identified by ClueGO into a final list of 19 biological themes based on the “GOGroups” column (Figure 5C and Supplemental Table 3). The most statistically significant of these biological themes was innate immune response, followed by defense response to virus (adjusted *P* values of 1.35 × 10^–35^ and 5.75 × 10^–31^, respectively).

We next utilized a clustered heatmap algorithm using the Seaborn package within Python to group the list of 19 biological themes based on the normalized expression of their cognate member genes. All the GOBP themes showed a low level of expression in baseline iCD103^+^ cells, which was greatly augmented following γHV-68 stimulation (Figure 5C). When expression levels were analyzed in γHV-68–treated iCD103^+^ cells treated with kyn, we noted a more muted expression profile with all the themes exhibiting a lower average expression of the associated genes (Figure 5C).

Cluster analysis revealed 3 distinct clades of GOBP themes with cluster 3 exhibiting the largest differences between iCD103^+^ DCs treated with kyn + γHV-68 and iCD103^+^ DCs treated with γHV-68 (Figure 5C, clade c3). This clade consisted of 3 GOBP themes, namely, cytokine activity, regulation of vasculature development, and leukocyte chemotaxis. Examination of the genes within the 3 categories in clade c3 revealed limited overlap between the 3 with vasculature development showing the most unique gene signature (Supplemental Figure 3A). We next utilized a clustered heatmap algorithm to group the 43 genes that comprised the 3 GOBP themes from Figure 5C, clade c3. Clustering analysis split these 43 genes into 4 distinct clades based on transcript expression (Supplemental Figure 3B). Clade c1 mostly comprised genes unique to the GOBP theme cytokine activity, which all had very low levels of expression in unstimulated (vehicle-treated) iCD103^+^ cells. Interestingly this clade comprised several genes identified previously in the qRT-PCR and RNA-Seq differential expression analysis, namely, IL-27, DLL4, and IFN-β1. Clades c2 and c4 had increased baseline expression in comparison with clade 1 but lower than clade 3. Clade c2 mostly comprised genes in the vasculature development GOBP theme, while clade 4 comprised just 2 related genes, CXCL9 and CXCL10, both of which exhibited increased expression in γHV-68–treated iCD103^+^ cells, which decreased in iCD103^+^ cells treated with kyn (2.9-fold and 2.8-fold downregulated, respectively, Supplemental Figure 3B). Finally, clade 3 exhibited much greater overall expression in vehicle-treated iCD103^+^ cells. Interestingly, 3 genes in clade 3 (*Alox5*, *Ptger3*, and *Pgf*) exhibited a unique gene expression pattern showing reduced expression in γHV-68–treated cells, which increased upon stimulation with kyn (2.5-fold, 2.6-fold, and 2-fold upregulated, respectively).

**Figure 6:**
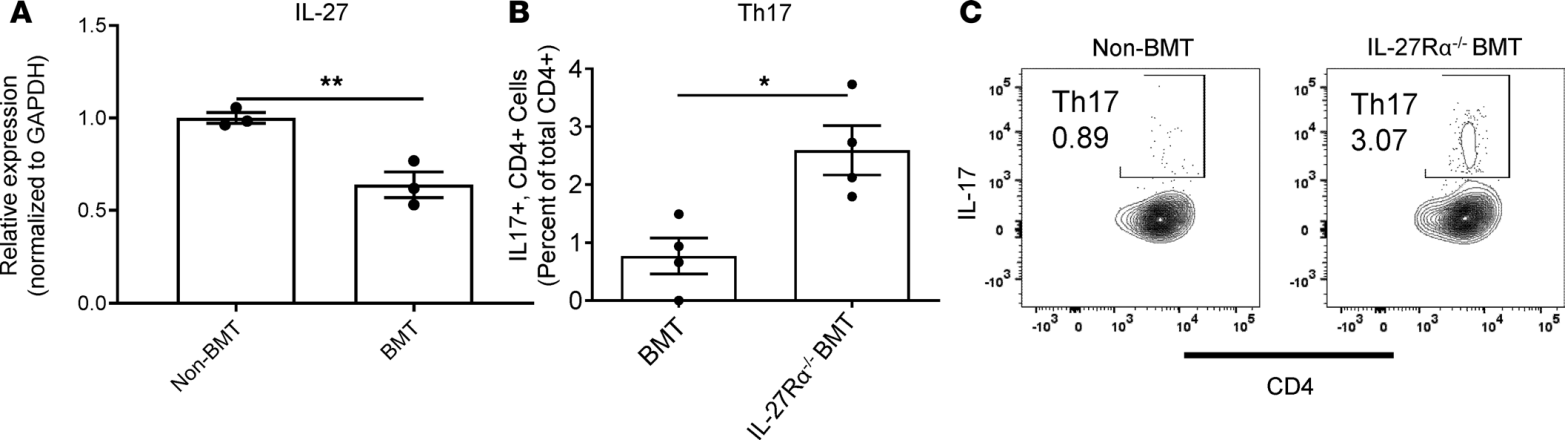
Loss of IL-27 enhances Th17 differentiation following BMT. (**A**) Total RNA was extracted from either non-BMT or BMT whole-lung homogenate (shown as mean ± SEM, *n* = 3 mice per group). Expression of the indicated transcript was assessed via qRT-PCR. (**B**) Number of Thy1.2^+^CD3^+^CD4^+^IL-17^+^ lymphocytes was assessed by flow cytometry with intracellular cytokine staining (ICS) in the lungs of non-BMT or BMT mice following γHV-68 infection (7 dpi, shown as mean ± SEM, *n* = 4 mice per group). Leukocytes from collagenase-digested lungs were first stimulated with PMA and ionomycin before staining for flow cytometry. (**C**) Representative plots from **B**. Statistical significance was determined by Student’s *t* test, *P < 0.05, **P < 0.01.

To experimentally validate some of the findings from our RNA-Seq data set, we next addressed the effects of 1 gene identified as being differentially regulated by CD103^+^ DCs in the presence of kyn, namely IL-27, utilizing IL-27Rα^–/–^ mice as BMT donors. As anticipated from the iCD103^+^ DC work in vitro, expression of IL-27 was decreased in the lungs of BMT mice following γHV-68 infection (Figure 6A). Moreover, differentiation of Th17 cells was especially pronounced when IL-27 signaling was further reduced post-BMT by using IL-27Rα^–/–^ bone marrow as a donor source (Figure 6B, representative flow plots shown in Figure 6C). Thus, these data establish IL-27 as an important suppressor of Th17 differentiation in response to respiratory herpesvirus infections following BMT.

### Ablation of IDO does not alter Th17 differentiation or IL-17 production in response to γHV-68 following BMT.

**Figure 7:**
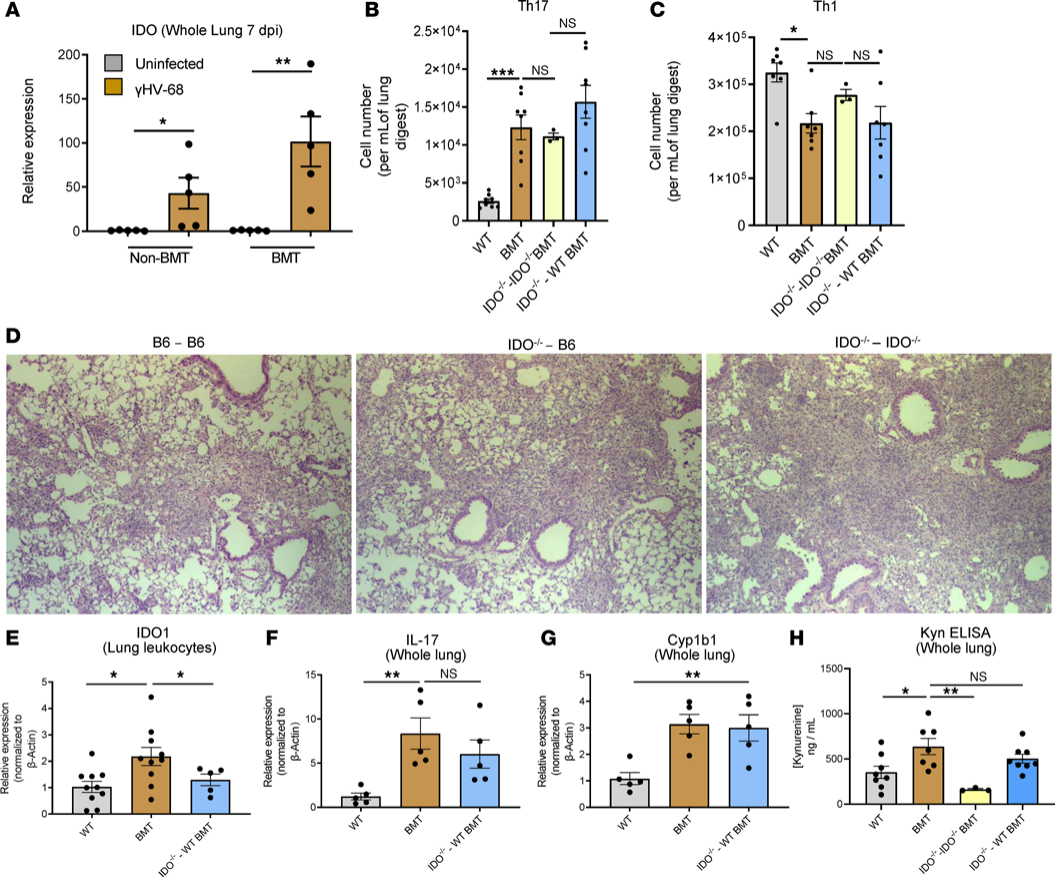
Ablation of IDO does not alter Th17 differentiation, IL-17 production, or fibrotic pathology in response to γHV-68 following BMT. (**A**) Groups of mice were analyzed for expression of IDO in whole-lung homogenate 7 days following γHV-68 infection (shown as mean ± SEM, *n* = 5 mice per group). (**B** and **C**) Lung leukocytes were isolated from collagenase-digested lung 7 days following γHV-68 infection (shown as mean ± SEM, WT, BMT, and IDO^–/–^ — BMT *n* = 8 mice per group, IDO^–/–^ — IDO^–/–^ *n* = 3 mice per group; the dashes signify the BMT pairing that was performed, with the donor cells denoted to the left of the dash and the recipient mouse strain on the right). Numbers of Th17 and Th1 cells were analyzed by ICS and flow cytometry. (**D**) Representative micrographs of sectioned lung tissue from the indicated transplant, stained with H&E. Original magnification, ×10. (**E**–**G**) Expression of indicated transcript was analyzed in lung leukocytes obtained from groups of mice at 7 dpi by qRT-PCR (shown as mean ± SEM, panel **E** WT *n* = 10, BMT, *n* = 10, IDO^–/–^ — BMT *n* = 5. Panels **F** and **G** *n* = 5 mice per group). (**H**) Concentration of kyn was analyzed in whole-lung homogenates at 7 dpi via ELISA (shown as mean ± SEM, WT, BMT, and IDO^–/–^ — BMT *n* = 8 mice per group, IDO^–/–^ — IDO^–/–^ *n* = 3 mice per group). Statistical significance was determined by ANOVA, *P < 0.05, **P < 0.01, ***P < 0.001.

AHR ligands, such as kyn, are mainly produced by the enzymatic processing of tryptophan beginning with the heme-dependent oxygenases IDO1 and TDO2. IDO1 expression can be induced in a number of inflammatory cell types, such as macrophages and DCs, and thus we first sought to characterize expression of IDO in BMT mice in response to γHV-68. Non-BMT mice infected with γHV-68 exhibited an increase in IDO expression at 7 dpi; however, expression of IDO1 was further augmented in infected BMT mice (Figure 7A). We further characterized the localization of IDO in BMT mice via immunofluorescence microscopy. In control lungs, IDO1 expression was restricted to CD68^+^ cells (most likely alveolar macrophages) as well as in airway epithelial cells and vascular smooth muscle cells (Supplemental Figure 4A). We did not detect appreciable colocalization of IDO in myofibroblasts expressing α–smooth muscle actin in the fibrotic areas of infected BMT mice, nor did we detect expression of IDO1 in type 1 or type 2 alveolar epithelial cells (T1α– or pro-surfactant protein C–expressing cells, respectively) (Supplemental Figure 4, B and C). In agreement with qRT-PCR data, IDO expression was greatly increased in the lungs of infected BMT mice with the majority of expression in airway epithelial cells and CD45^+^ cells in infected BMT mice (Supplemental Figure 4, D and E).

We next sought to ascertain the importance of IDO1 on the differentiation of T cells by utilizing IDO^–/–^ mice as donors and/or recipients for BMT. Interestingly, even though there was an approximately 100-fold increase in IDO1 expression in the lungs of BMT mice in response to γHV-68 (Figure 7A), IDO^–/–^ into IDO^–/–^ or IDO^–/–^ into B6 BMT mice showed no difference in numbers of either Th17 or Th1 cells present at 7 dpi (Figure 7, B and C). Further, there was no difference in pathology by histological analysis (Figure 7D). To verify that expression of IDO was indeed diminished in leukocytes from IDO^–/–^ into B6 BMT mice, leukocytes were purified from collagenase-digested lungs, and expression of IDO was analyzed by qRT-PCR (Figure 7E). We further verified that expression of IL-17 was unchanged in IDO^–/–^ into B6 BMT mice by qRT-PCR of whole-lung homogenate (Figure 7F). Further, expression of the AHR-responsive gene *Cyp1b1* and concentration of kyn were not altered in the lungs of IDO^–/–^ into B6 BMT mice in comparison with WT-BMT mice; however, there was a significant decrease in lung-associated kyn in IDO^–/–^ into IDO^–/–^ BMT mice (Figure 7, G and H, respectively). Taken together with the results in the IDO^–/–^ into IDO^–/–^ BMT mice (Figure 7, B–D), these data indicate an alternative, non–IDO-dependent, pathway for the generation of fibrosis following BMT.

### TDO2 is upregulated in BMT fibrosis, and inhibition of TDO2 decreases collagen accumulation.

**Figure 8:**
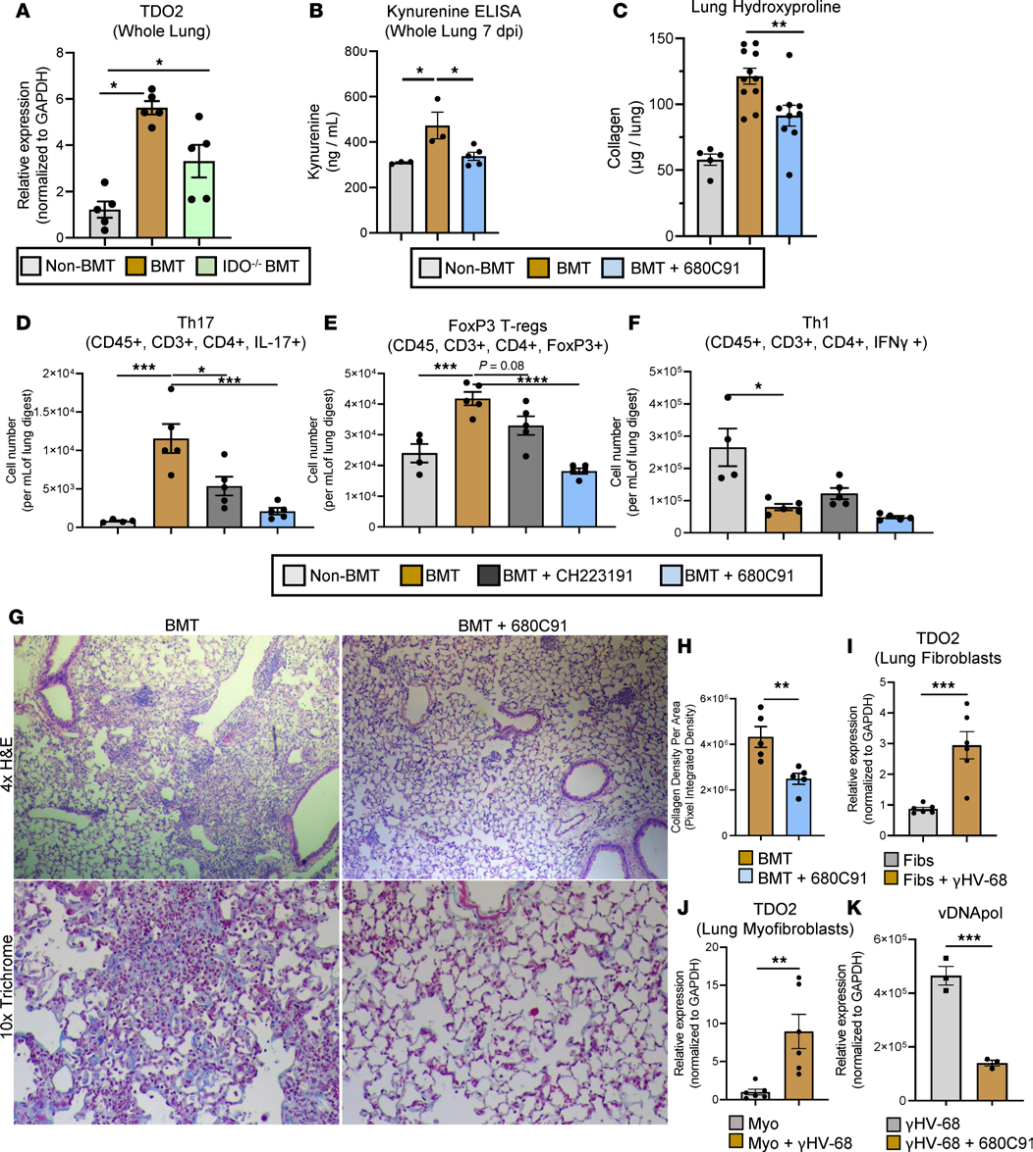
TDO2 is upregulated in BMT fibrosis, and inhibition of TDO2 ameliorates post-BMT pathology and alters lymphocyte skewing. (**A**) Groups of non-BMT (*n* = 5), BMT (*n* = 5), or IDO^–/–^ BMT (*n* = 5) mice were infected for 7 days with γHV-68. Lungs were collagenase digested and expression of TDO2 was analyzed by qRT-PCR. (**B** and **C**) Groups of non-BMT or BMT mice were infected with γHV-68 and treated with the TDO2-specific inhibitor 680C91 (10 μg per mouse every 2 days) for 7–14 days by i.p. injection. At 7 dpi, concentration of kyn was assessed from whole-lung homogenate via ELISA (**B** shown as mean ± SEM, non-BMT and BMT groups *n* = 3, BMT + 680C91 *n* = 5 mice per group). At 21 dpi, lungs were harvested, and lung collagen content was assessed by hydroxyproline assay (**C** shown as mean ± SEM, non-BMT *n* = 5, BMT *n* = 11, BMT + 680C91 *n* = 9). (**D**–**F**) Flow cytometry analysis of lung leukocytes harvested at 7 dpi and analyzed for the indicated lymphocyte population (shown as mean ± SEM, non-BMT *n* = 4, BMT *n* = 5, CH223191 [AHR antagonist] *n* = 5, 680C91 [TDO2-specific inhibitor] *n* = 5, both given at doses of 10 μg per mouse every other day). (**G**) Representative micrographs of lung sections taken from BMT mice infected with γHV-68 for 21 days. (**H**) Collagen fibers of micrographs from trichrome-stained lung sections were quantified in ImageJ (NIH; shown as mean ± SEM, *n* = 5 random fields per image). (**I**–**K**) Fibroblasts were isolated via “crawl-out” method from minced lung. Fourteen days after isolation, cells were infected with γHV-68 (MOI = 1.0) or mock infected for 24 hours, after which expression of the indicated transcript was analyzed by qRT-PCR (shown as mean ± SEM, *n* = 6 samples per group in **I** and **J**, *n* = 3 samples per group in **K**). Statistical significance was determined by ANOVA in panels **A**–**F** and Student’s *t* test in panels **H**–**K**, *P < 0.05, **P < 0.01, ***P < 0.001, ****P < 0.0001.

TDO2 is a heme-dependent oxygenase that, like IDO1, is an important rate-limiting enzyme for the conversion of tryptophan along the KP. TDO2 is highly expressed in liver; however, we believe its role in viral respiratory infections is unstudied. We examined expression of TDO2 in the lungs of non-BMT and BMT mice infected with γHV-68. We observed an approximately 6-fold increase in TDO2 expression at 7 dpi, and expression of TDO2 was also elevated in IDO^–/–^ into IDO^–/–^ BMT mice (Figure 8A). We next asked whether inhibition of TDO2 could rescue fibrotic pathology. To address this, groups of mice were infected i.n. with γHV-68 and treated with a specific TDO2 inhibitor, 680C91, or with vehicle control. Inhibition of TDO2 significantly reduced kyn concentration in the lungs of infected BMT mice at 7 dpi (Figure 8B). Further, there was a reduction in collagen accumulation in the lung as assessed by hydroxyproline assay at 21 dpi (Figure 8C). Interestingly, significantly fewer Th17 and FoxP3^+^ lymphocytes were detected in the lungs of 680C91-treated mice at 7 dpi with similar trends also noted for mice treated with the AHR inhibitor CH223191; however, no change in IFN-γ–secreting Th1 cells was detected in either inhibitor-treated group in comparison with vehicle-treated BMT mice (Figure 8, D–F). Additionally, we detected fewer IL-10^+^CD4^+^ lymphocytes (Tr1 cells) in the lungs of BMT mice, and BMT mice had a much lower Tr1/Th17 ratio in comparison with non-BMT mice (data not shown and Supplemental Figure 5). Inhibition of TDO2 partially restored the Tr1/Th17 ratio, indicating that blockade of TDO2 limits Th17 differentiation and increases Tr1 differentiation, possibly through modulation of DC IL-27 secretion.

We further observed less pathology via histological examination of H&E-stained lung slices and less deposition of collagen by trichrome staining (Figure 8G, quantified in Figure 8H). Because we delivered γHV-68 via an i.n. route, the likely first sites of viral replication are thought to be either lung epithelial cells or potentially fibroblasts. Thus, we examined expression of TDO2 in primary lung alveolar epithelial cells (AECs), primary lung fibroblasts, and a murine lung epithelial cell line (MLE12). Expression of TDO2 was not altered in primary AECs or MLE12 cells infected with γHV-68 and in fact was slightly repressed following infection (Supplemental Figure 6, A and B). We also investigated the effects of inhibition of TDO2 on viral replication by treating infected MLE12 epithelial cells with 680C91 but observed no difference in expression of the viral DNA polymerase, vDNApol (Supplemental Figure 6C). In contrast, TDO2 expression was elevated in primary lung fibroblasts following γHV-68 infection (Figure 8I) and to a greater extent in lung fibroblasts that had been differentiated into profibrotic myofibroblasts by culturing for 48 hours with recombinant TGF-β (Figure 8J). Further, counter to what was observed in epithelial cells, treatment of primary fibroblasts with the TDO2 inhibitor 680C91 greatly reduced viral gene expression at 24 hours postinfection while infection of fibroblasts from IDO^–/–^ mice resulted in similar expression of viral genes in comparison with WT (Figure 8K and data not shown). Together these data indicate a specific role for TDO2 in augmenting viral replication in fibroblasts but not epithelial cells.

## Discussion

Pulmonary complications are a major cause of post–hematopoietic stem cell transplant morbidity and mortality in both allogeneic and autologous stem cell transplantation (27, 28). Further, reactivation of herpesviruses is common following transplant, and herpesvirus infections are strongly associated with the development of deadly post-BMT pulmonary complications (1, 29, 30). Thus, understanding the complex nature of the altered host-pathogen interaction that occurs in the context of the lung following transplant is paramount to developing new therapeutics. Here, we have utilized a model of syngeneic BMT and herpesvirus infection that allows us to study these important immunological alterations without the confounding influence of common postallogeneic complications, such as graft-versus-host disease. We have demonstrated a role for DC AHR signaling in the exacerbation of virally induced post-BMT pneumonitis and fibrosis. Specifically, we observed that AHR^+^CD103^+^ DCs accumulate in the lungs following BMT in response to herpesvirus infection and respond to increased levels of the AHR ligand kyn, resulting in a skewing of the DCs toward a pro-Th17 phenotype.

Previous studies have demonstrated a role for AHR in controlling leukocyte cytokine responses, and AHR signaling can repress or activate Th17 differentiation in various contexts. Quintana et al. originally reported that stimulation of lymphocytes with dioxin augmented Treg but repressed Th17 differentiation; however, T cells stimulated with the endogenous AHR ligand FICZ had the opposite response and exhibited enhanced Th17 differentiation and experimental autoimmune encephalomyelitis pathogenicity (31). Furthermore, TCDD (dioxin) was found to ameliorate pathology in a murine colitis model through a similar mechanism of upregulated Treg differentiation (32). Interestingly, BMT mice infected with γHV-68 also exhibit elevated numbers of FoxP3^+^ Tregs in the lungs (33). Further, mice treated with either an AHR antagonist or a TDO2 antagonist had fewer FoxP3^+^ Tregs in the lungs following infection (Figure 8E). Together, these data are suggestive of a role for increased AHR signaling in the differentiation of Tregs following BMT.

The IDO/AHR axis was also recently shown to limit pulmonary inflammation following allogeneic BMT (10). This phenotype was shown to be T cell centric as purified T cells expressed less IL-17 and IFN-γ. In contrast to this study, we did not see any difference in cytokine production in the lungs of IDO^–/–^ into IDO^–/–^ BMT mice infected with γHV-68, nor did we see any difference in the numbers of AHR^+^ lymphocytes between WT and BMT mice (Figure 7, B–C, and Figure 3B, respectively). These discrepancies are most likely due to the differences between the nature of the inflammatory insult following BMT, i.e., sterile alloimmune reactions contrasted with viral infection.

In lieu of AHR^+^ lymphocytes, we instead saw an accumulation of AHR^+^CD103^+^ DCs, which were likely responding to kyn produced by TDO2-expressing fibroblasts. When iCD103^+^ DCs were preincubated with γHV-68 and kyn, which was subsequently washed out before coculture with T cells, we observed enhanced Th17 differentiation and production of IL-17 (Figure 4G); however, when iCD103^+^ DCs and T cells were cocultured together in the presence of kyn, we noticed an inhibitory effect on IL-17 production (data not shown), indicating that kyn is a pluripotent immune modulator and can augment or repress IL-17A production, via AHR signaling in DCs or lymphocytes, respectively. Additionally, alterations in cytokine output from iCD103^+^ cells happened only in the presence of both viral and AHR stimulation. This is most likely due to the interactions of the AHR pathway with inflammatory signaling pathways, e.g., TLR signaling, induced during viral recognition by DCs.

AHR has also previously been shown to modulate DC function, and the interaction of AHR with activating ligands, such as the kyn family of tryptophan metabolites, has been characterized to influence both the maturation and function of DCs in various models. Treatment of bone marrow–derived DCs (BMDCs) with the potent AHR ligand, dioxin, upregulated expression of MHCII and the costimulatory protein CD86 but decreased production of IL-10 (34). More recently, treatment of BMDCs with the endogenous AHR ligand, FICZ, similarly upregulated expression of MHCII, CD86, and CD11c and augmented expression of IL-6 (35). Cotreatment of DCs with AHR-activating, air pollution–derived particulate matter (PM_2.5_) augmented expression of the pro-Th17 cytokines IL-6, IL-1β, and IL-22 in an AHR-dependent manner (36). Further, AHR signaling has been shown to modulate DC responses to TLR stimulation. Specifically, coadministration of the AHR ligand TCDD (dioxin) augmented expression of some cytokines and chemokines, i.e., IL-1β and CCL1, in response to LPS stimulation while repressing expression of others (19). We noted a similar effect of AHR stimulation on iCD103^+^ DC expression of cytokines in response to γHV-68 stimulus in that expression of some cytokines, notably IL-27, was repressed while expression of other cytokines, e.g., IL-23, was maintained (Figure 4). However, in contrast to what was noted in previous studies using GM-CSF–derived BMDCs, we noted decreased expression of IL-6 and IL-1β in iCD103^+^ DCs (Figure 4, Figure 5, and Supplemental Table 1).

We also noted decreased expression of genes important in recruiting and maintaining Th1 lymphocytes in iCD103^+^ DCs treated with kyn, namely, CXCL9, CXCL10, IL-27, and the notch ligand DLL4 (37, 38). Interestingly, we noted marked upregulation of several genes involved in eicosanoid signaling, namely, arachidonate 5-lipoxygenase (Alox5) and prostaglandin E2 receptor 3 (Ptger3). Prostaglandin signaling through the Ptger family receptors has been directly implicated in the maintenance of pro-Th17 DC cytokines, such as IL-23 (39–41). Further, Alox5 is a critical enzyme in the synthesis of leukotrienes, which have also been implicated in the skewing and maintenance of Th17 cells (42, 43). Thus, our data indicate that activation of AHR in CD103^+^ DCs by tryptophan metabolites produced during inflammatory insult result in expression of pro-Th17 genes and reduction in expression of pro-Th1 genes that contributes to the fibrotic pathology noted in BMT mice. Further, our data and the previously mentioned studies highlight the role of AHR in modulating DC cytokine responses and demonstrate that AHR signaling in DCs provides a mechanism to fine-tune immune responses to various stimuli rather than acting as an on/off switch.

Interestingly, we noted increased expression of both IDO1 and TDO2 in the lungs of BMT mice. However, genetic ablation of IDO1 on hematopoietic and/or structural cells in the lung did not influence the generation of fibrosis in BMT mice while inhibition of TDO2 did (Figure 7 and Figure 8, respectively). This finding was surprising because expression of IDO1 and IDO2 but not TDO2 were shown to be increased, specifically, in the lung following inflammatory (LPS) stimulation (44). Additionally, Lee et al. demonstrated a crucial role for IDO in limiting pulmonary inflammation following allogeneic BMT, where IDO expression was localized to lung epithelial cells (10). Although we were able to localize IDO expression to airway epithelial cells and leukocytes in the lungs following herpesvirus infection in BMT mice (Supplemental Figure 3), IDO was not expressed in AECs or myofibroblasts, both cell types that are critically important in the pathogenesis of fibrosis. Ablation of all IDO expression, i.e., IDO^–/–^ into IDO^–/–^ BMT mice, did exhibit a significant decrease in lung-associated kyn; however, this did not translate into alterations in T cell differentiation or cytokine expression (Figure 7).

In contrast, pharmacological inhibition of TDO2 decreased numbers of Th17 cells and increased the Tr1/Th17 ratio in the lungs following infection (Figure 8, Supplemental Figure 5). The restoration of Tr1 differentiation is especially intriguing because IL-27 and type I IFNs, which were both decreased in iCD103^+^ DCs treated with kyn, have been shown to be critically important for the differentiation of IL-10–producing Tr1 cells (45, 46). Although AHR signaling has also been implicated in the differentiation of Tr1 cells, the increased kyn production observed in BMT mice was inversely correlated with numbers of Tr1 cells (47). This observation, coupled with the disparity between IDO1 and TDO2 functionality following BMT, indicate that factors such as cellular compartmentalization, i.e., where the kyn is produced, and the nature of the inflammatory insult, i.e., infectious versus noninfectious, most likely play important roles in dictating which cells receive stimulus through AHR and how those signals are interpreted.

**Figure 9:**
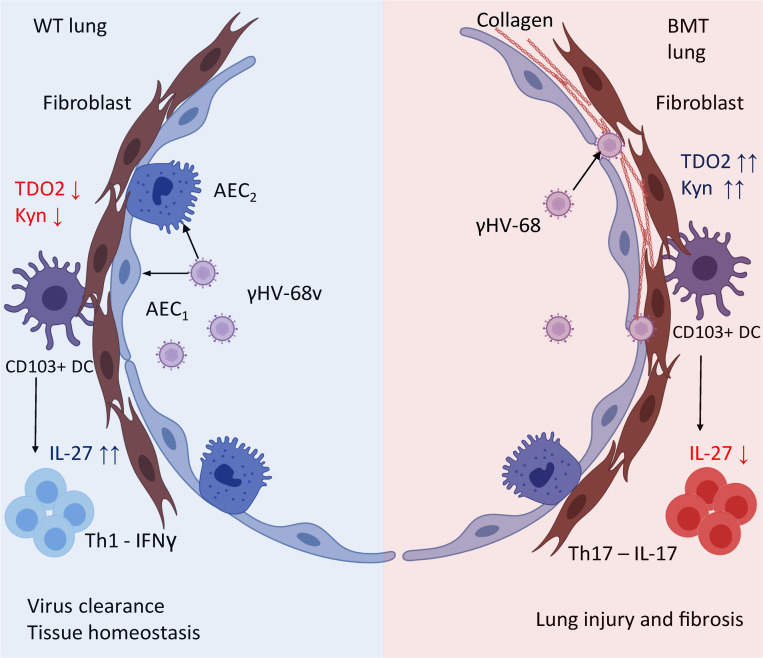
Hypothesized model of lung injury driven by TDO2–AHR following BMT. In non-BMT (WT) lungs, viral replication is largely localized to the alveolar epithelium, which results in decreased expression of TDO2 and lower kyn levels, allowing for production of IL-27 by lung-resident CD103^+^ DCs, resulting in a Th1-skewed microenvironment that aids in viral clearance. In BMT lungs, viral infection of lung fibroblasts drives production of TDO2 and increases kyn levels, which suppresses IL-27 from CD103^+^ DCs, resulting in increased Th17 differentiation, lung injury, and fibrosis.

TDO2 expression has not been well characterized in the lung and has only been described in lung fibroblasts in a lung cancer model (48). Thus, to our knowledge, we are the first to report increased TDO2 expression following viral infection in the lung. We further demonstrated that TDO2 expression was important for herpesvirus replication in fibroblasts but not epithelial cells (Figure 8), indicating a cell-specific effect of TDO2 action on viral replication. Our current working hypothesis is that increased activation of the KP drives profibrotic IL-17 responses via upregulation of TDO2 in infected fibroblasts in the fibrotic niche created in BMT mice (Figure 9). AHR^+^CD103^+^ DCs seem uniquely poised in the lung to integrate AHR stimulation, which can in turn modulate cytokine output and drive pulmonary pathology. Thus, the fibroblast (TDO2)/DC (AHR) axis is an interesting and potentially novel pathway for intercellular communication between nonimmune cells and leukocytes that might be exploited as a therapeutic target for pulmonary inflammation and fibrosis. We find it particularly interesting that kyn derived from IDO in other cell types (e.g., airway epithelium and leukocytes) is unable to have the same impact on AHR^+^CD103^+^ DC function and subsequent Th17 responses, suggesting the importance of specific fibroblast/DC communication in regulating outcomes. Future studies are needed to fully understand how kyn (or other TDO2-derived metabolites) differentially regulate herpesvirus replication in fibroblasts versus epithelial cells.

## Methods

### Mouse strains, cell lines, cell isolations, and virus.

Male C57BL/6J mice were purchased from The Jackson Laboratory; AHR^–/–^ mice were purchased from Taconic (Bio-Techne, mouse 9166). All mice used were between 6 and 8 weeks of age at the beginning of experiments and housed in specific pathogen–free conditions at the University of Michigan. BMTs were conducted as previously described (49). In short, recipient mice were irradiated with 13.5 Gy (split dose) from a ^137^Cs source. Following irradiation, 5 × 10^6^ donor bone marrow stem cells (either syngeneic stem cells from C57BL/6J mice or congenic knockout stem cells from a C57BL/6J background) were injected via the tail vein. Mice were subsequently housed for 5 weeks for hematopoietic reconstitution before infection with 5 × 10^4^ PFU γHV-68 VR-1465, purchased from ATCC, via i.n. inoculation. Experiments were then performed at days 7 and 21 postinfection.

γHV-68 (VR-1465) was propagated on NIH3T12 (CCL-164) cells purchased from ATCC. Primary fibroblasts were obtained from the lungs of naive mice by crawl-out method from lung minces. Lungs were first aseptically minced with scissors before plating in a cell culture flask with complete DMEM culture media containing 10% FBS, and 100 U each of penicillin and streptomycin, for 14 days. Primary AECs were isolated as previously described (50). In short, lungs were cannulated via the trachea and cast with dispase (Corning) and low-melting-temperature agarose (Thermo Fisher Scientific) before incubation in dispase solution. Hematopoietic cells were excluded by CD45^+^ magnetic bead selection (CD4^+^ beads L3T4, Miltenyi Biotec), and fibroblasts and macrophages were removed by adherence purification. The resulting cells were plated on fibronectin-coated plates and allowed to differentiate for 2 days before further experimentation. Epithelial cells were cultured in Dulbecco’s modified essential media supplemented with 10% heat-inactivated FBS, pen/strep, and amphotericin B. Primary naive CD4^+^ T cells were isolated from the spleens of C57BL/6J mice by magnetic bead purification and cultured in RPMI-1640 (HyClone, Cytiva) supplemented with 10% heat-inactivated FBS, pen/strep, and amphotericin B.

ICD103^+^ cells were cultured as previously described (23). In short, bone marrow stem cells were aseptically isolated from the femurs of 6- to 8-week-old C57BL/6J mice and cultured for 9 days in RPMI-1640 medium with the addition of 10% FBS, pen/strep cocktail, 50 μM β-mercaptoethanol, 5 μg/mL recombinant murine GM-CSF, and 200 μg/mL recombinant murine FLT3L (both purchased from R&D Systems, Bio-Techne). On the ninth day of culture, floating cells were replated in the same supplemented media and cultured for an additional 7 days. At day 16 after isolation, cells were used in downstream experiments.

### AHR pathway cell culture reagents.

Kyn was purchased from MilliporeSigma and used at a final concentration of 200 μM. The AHR and TDO2 inhibitors CH223191 and 680C91 were purchased from R&D Systems (Bio-Techne) and were used at a concentration of 10 μM for in vitro experiments. For in vivo administration of CH223191 and 680C91, mice received 10 μg of either drug in a vehicle of 2% Tween-20 in sterile PBS via i.p. injection starting 1 day after infection with γHV-68 and given every other day until the day of harvest.

### Gene expression and cytokine quantitation via qRT-PCR, RNA-Seq, and ELISA.

Total RNA was extracted via TRIzol reagent (Thermo Fisher Scientific) following the manufacturer’s direction. Gene expression was analyzed via quantitative PCR using a Taqman RNA-to-CT real-time master mix kit (Thermo Fisher Scientific) and was analyzed on an ABI StepOnePlus thermocycler (Thermo Fisher Scientific). For RNA-Seq, RNA was extracted using a RNeasy column kit (Qiagen) following the manufacturer’s directions. RNA integrity was assessed on an Agilent Bioanalyzer, and all RNA integrity values were assessed at 8 or higher before RNA sequence analysis. Cytokine and metabolite concentrations were determined from either cell culture supernatant or whole-lung homogenate with the following: IL-17A DuoSET (R&D Systems, Bio-Techne) and kyn ELISA (Rocky Mountain Diagnostics). All assays were conducted according to manufacturers’ directions. Primers used for qRT-PCR are detailed in Table 1. RNA-Seq was performed by Genewiz using its standard RNA-Seq pipeline. In short, sequence reads (30 million–50 million read depth) were trimmed to remove possible adapter sequences and nucleotides with poor quality using Trimmomatic v.0.36. Reads were mapped to the Ensembl mouse reference genome using the STAR aligner v.2.5.2b. Unique gene hit counts were calculated by using featureCounts from the Subread package v.1.5.2. Only unique reads that fell within exon regions were counted. Transcript counts were normalized, and differential expression statistics were calculated using DESeq2. RNA-Seq data were visualized using Python 3.7 and a combination of Matplotlib, Scikitlearn, and Seaborn packages. Both raw and processed RNA-Seq data are available in total through the NCBI’s Gene Expression Omnibus database, accession number GSE160307.

### Collagenase digestion of lungs and isolation of lung leukocytes for flow cytometry.

Primary lung leukocytes were obtained by collagenase digestion of whole lung as previously described (51). In short, lungs were digested in complete DMEM containing 15 mg/mL collagenase A (Roche) and 2500 U of DNase I (MilliporeSigma) for 30 minutes at 37°C. Digested tissue was mechanically disrupted, and leukocytes were purified by centrifugation (20 minutes at 2000*g*, 4°C) through a 20% Percoll solution. For some experiments, ICS was performed. Cells were diluted to 1 × 10^6^/mL and stimulated for 4 hours with PMA (10 ng/mL), ionomycin (10 μM), and GolgiStop reagent (BD Biosciences). Antibodies used for flow cytometry were as follows: myeloid flow cytometry staining panel, BV650-CD11b (clone M1/70), BV510-CD45 (clone 30-F11), BV421-I-Ab (MHCII clone AF6-120.1), APC-Cy7-SiglecF (clone E50-2440), APC-Ly6G (clone 1A8) purchased from BD Horizon. BV605-CD64 (clone X54-5/7.1), PerCP-Cy5.5-CD24 (clone M1/69), PE-Cy7-Ly6C (clone HK1.4), PE-CD193 (CCR3 clone J073E5) purchased from BioLegend. PE-eFluor610-CD11c (clone N418), PE-AHR (clone 4MEJJ) purchased from eBioscience, Thermo Fisher Scientific. Lymphocyte flow cytometry staining panel, PE-Cy7–IFN-γ (clone XMG1.2), FITC-CD3 (clone 17A2) purchased from BD Biosciences. APC-Cy7-IL17A (clone TC11-18H10.1), BV570-CD8a (clone 53-6.7), AF700-CD90.2 (Thy1.2 clone 30-H12), BV510-CD4 (clone GK1.5) purchased from BioLegend. PE-eFluor 610-FoxP3 (clone FJK-16s) purchased from eBioscience, Thermo Fisher Scientific.

### Statistics.

Statistical analyses were done using GraphPad Prism v8.0 (Graph Pad Software Inc.). To determine statistical significance, Student’s *t* test (2 tailed) was performed for all 2-group comparisons; ANOVA (1 way) was used when more than 2 groups were compared. All experiments were conducted in at least duplicate with similar results. *N* values and other statistical information are given in individual figure legends. Collagen fibers were quantified in ImageJ from trichrome-stained micrographs as described (52). In short, micrographs were first processed through a color deconvolution algorithm, after which, a threshold was set on the color channel representing the collagen staining, and pixel densities from 5 random fields of equal size were measured per image. *P* < 0.05 was considered significant.

### Study approval.

All animal experiments were conducted in accordance with an IACUC-approved animal protocol at the University of Michigan. Protocol PRO00008731, “Immunobiology of the Lung in Fibrosis and Transplant,” expires October 31, 2021.

## Author contributions

SJG designed and performed experiments, collected and analyzed data, and wrote and edited the manuscript. NLP and SMH performed experiments and analyzed data. CW performed experiments. RLZ and BBM designed experiments, analyzed data, and edited the manuscript.

## Supplementary Material

Supplemental data

Supplemental Table 1

Supplemental Table 2

Supplemental Table 3

## Figures and Tables

**Table 1:**
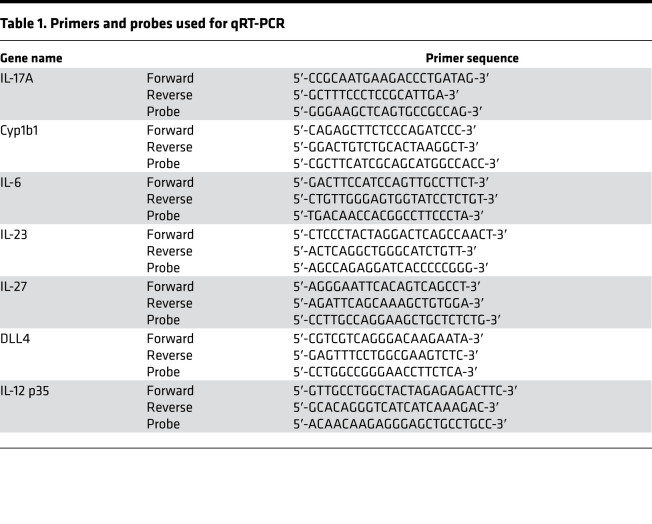
Primers and probes used for qRT-PCR

## Version 1 01/25/2021

Electronic publication
