# Supplementary material for: Stem cell transplantation uncovers TDO-AHR regulation of lung dendritic cells in herpesvirus-induced pathology
Source: JCI Insight. 2021 Jan 25;6(2):e139965. doi: 10.1172/jci.insight.139965 (PMC7934859; doi:10.1172/jci.insight.139965)
Supplement: Supplemental data [file jciinsight-6-139965-s020.pdf]

Figure S1

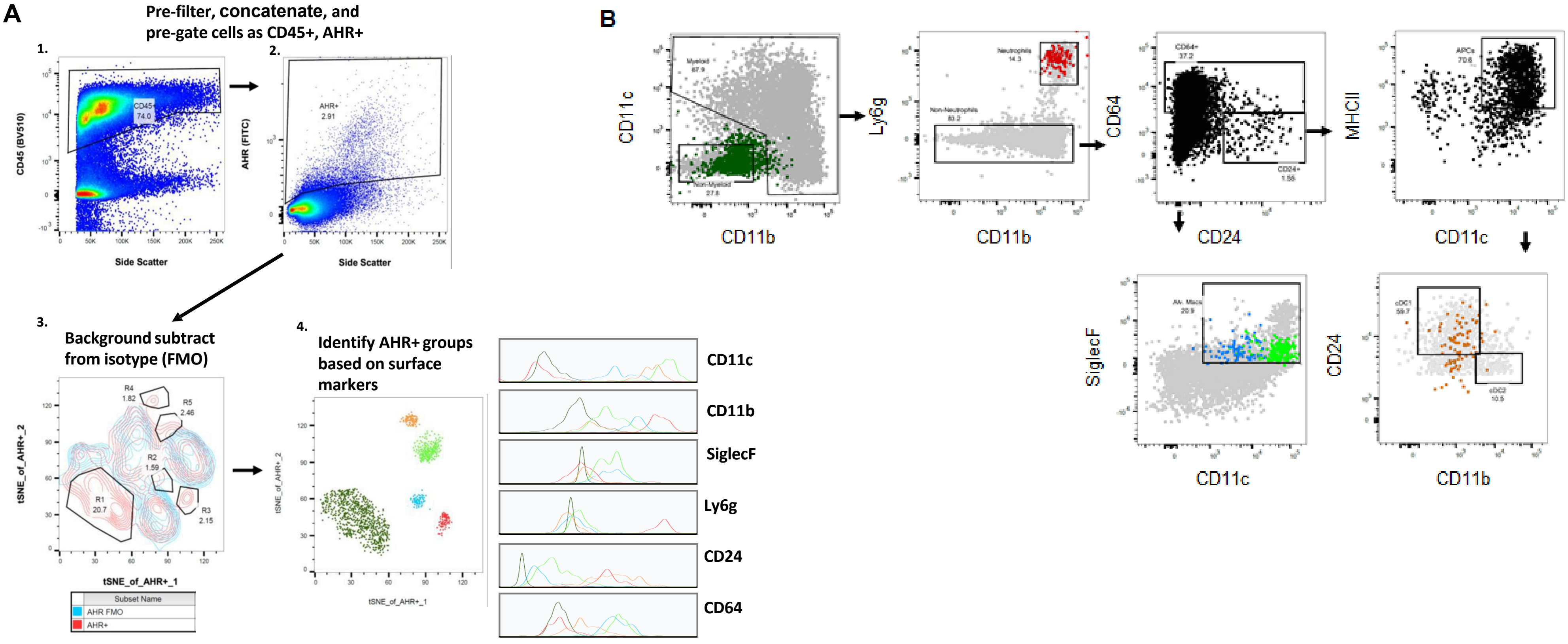

Figure S1. Flow gating scheme for tSNE analysis of AHR+ cells. A) Following 7 days of infection with γHV-68, lungs were harvested from groups of mice and leukocytes were purified by collagenase digestion. Leukocytes were stained with CD11c, CD11b, SiglecF, Ly6G, CD24 and CD64 and analyzed by flow cytometry. All samples were first concatenated together using Flowjo 10.5. Cells were next gated as being CD45+ and AHR+ (panels 1 and 2). Next, tSNE multidimensional reduction analysis was conducted on all AHR+ cells and background expression was corrected for by comparing samples against an isotype control (Panel 3). Finally, groups of AHR+ cells were identified by surface marker expression (Panel 4). B) AHR+ cells identified in panel A were backgated onto a traditional flow cytometry gating scheme. Cells were first gated CD45+ before gating on CD11b and CD11c (myeloid cells). Neutrophils were gated as being CD11b+ and Ly6g+. Macrophages were next gated as being CD64+ while APCs were gated as CD24+. Finally, CD64+ macrophages were additionally gated using SiglecF to identify alveolar macrophages while APCs were separated using CD24+ (cDC1) or CD11b+ (cDC2). All colors correspond to the colored groups in figure 3.

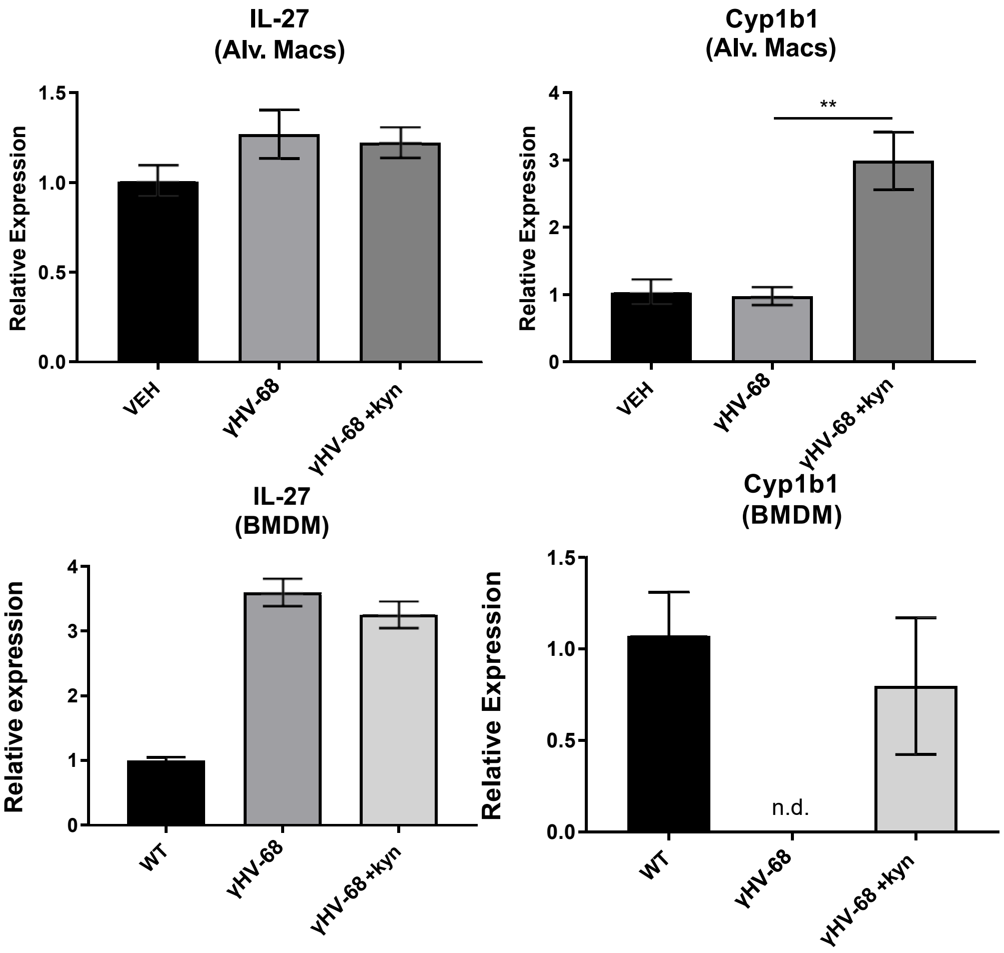

Figure S2. Kynurenine treatment does not alter expression of IL-27 in alveolar macrophage. Macrophage were isolated from the lungs of naïve mice by broncho-alveolar lavage. Cells, n = 3 samples per group, were infected with γHV-68 and treated with 200 μM kyn or vehicle for 24 h. Transcript expression was quantified by qRT-PCR. Statistical significance was calculated by ANOVA, ** = $p < 0.01$.

Figure S3

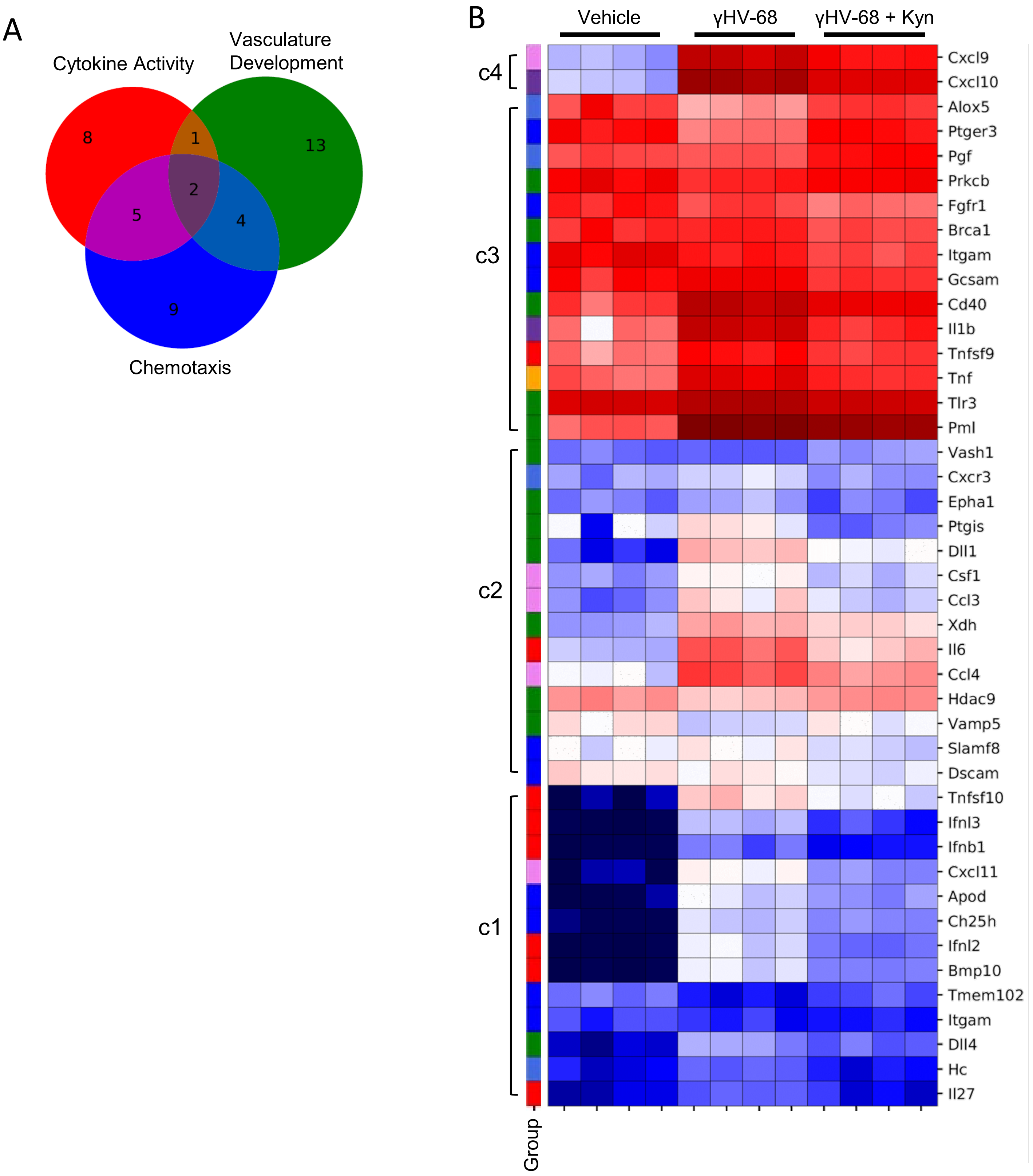

Figure S3. Cluster analysis of cognate genes from ontology themes (clade 3 from figure 5c). (A) Venn diagram showing the numbers of unique, and overlapping, genes comprising the three gene ontology themes identified in figure 5c clade 3. (B) Clustered heatmap analysis of genes identified in the three aforementioned GOBP themes. Side brackets denote distinct clades identified by the clustering algorithm. The multicolored squares on the left spine of the heatmap correspond to which group from panel (A) the gene belongs to.

Figure S4

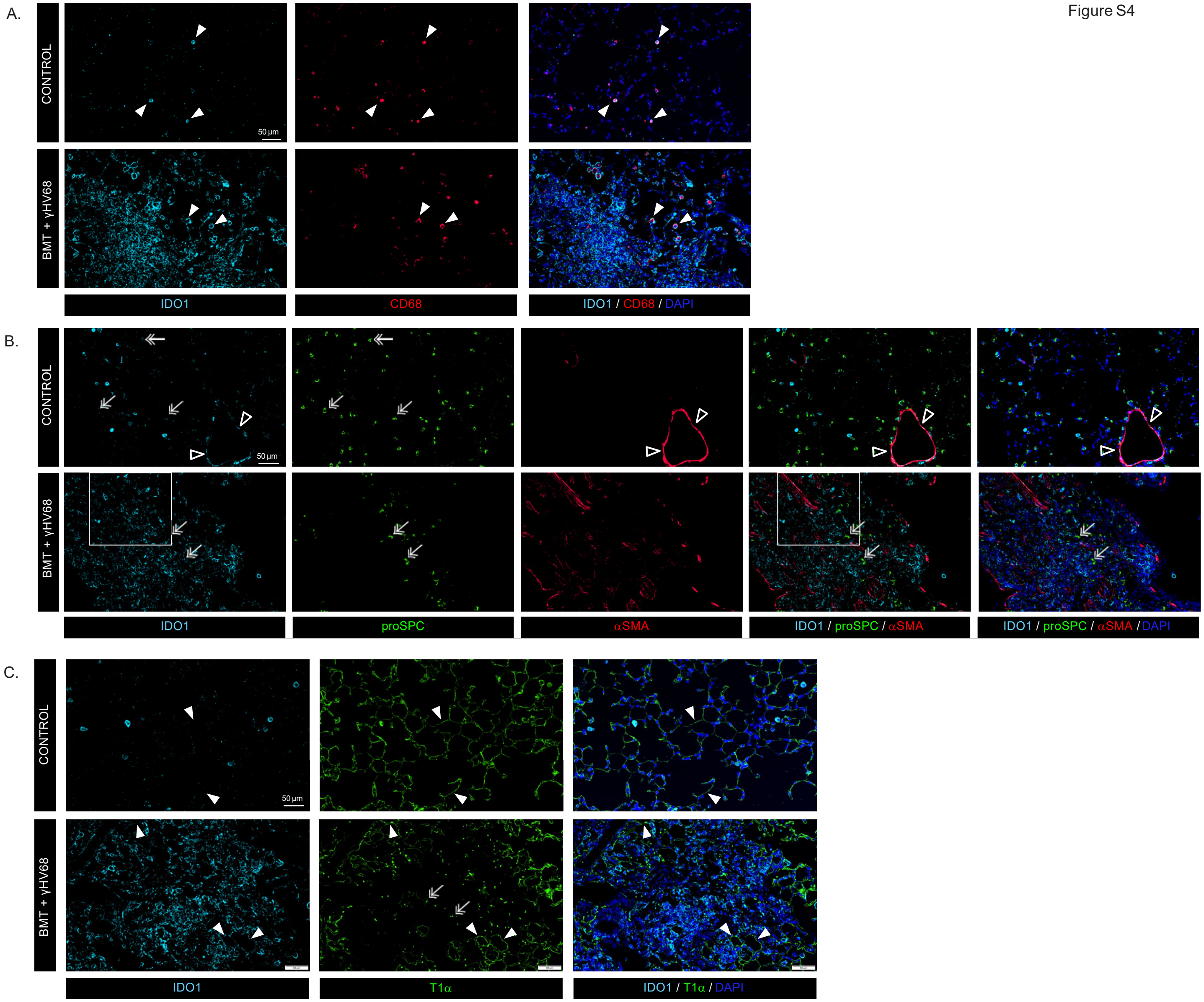
A.
B.
C.
CONTROL
BMT + γHV68
50 µm
IDO1
CD68
IDO1 / CD68 / DAPI
proSPC
αSMA
IDO1 / proSPC / αSMA
IDO1 / proSPC / αSMA / DAPI
T1α
IDO1 / T1α / DAPI

D.

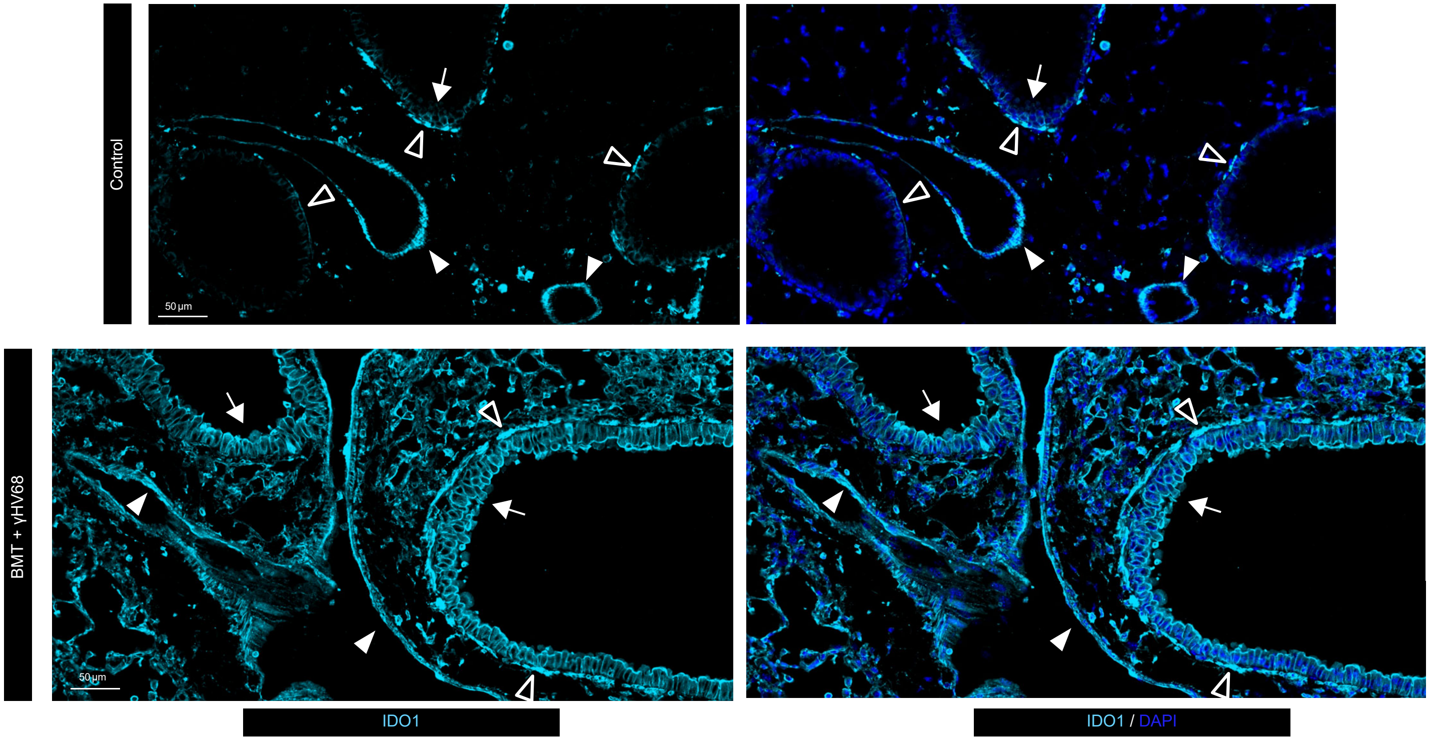

Control
BMT + γHV68
50 μm
50 μm
IDO1
IDO1 / DAPI

E.

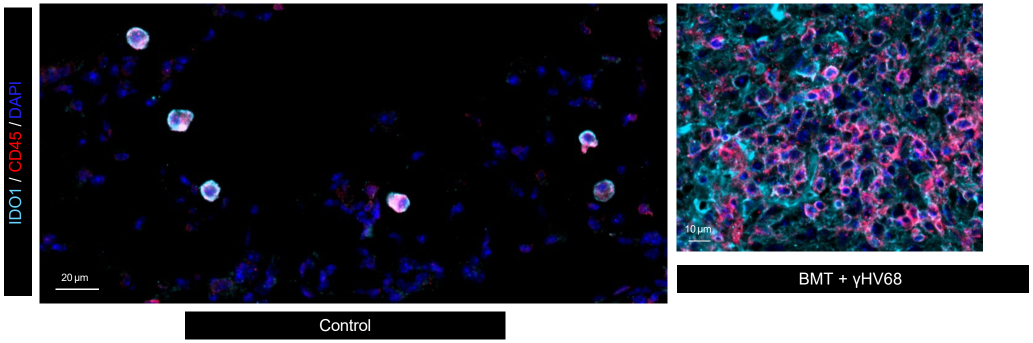

IDO1 / CD45 / DAPI
20 μm
10 μm
Control
BMT + γHV68

Figure S4. IDO1 Expression Increases after BMT + γHV68. Slides were deparaffinized and antigens were retrieves using DAKO target retrieval solution (DAKO, Agilent, Santa Clara CA). Slides were bocked in 5% normal donkey serum in TTBS before addition of appropriately diluted primary and secondary antibody cocktails. Primary antibodies used were as follows: anti-IDO1 (Santa Cruz, sc-53978), anti-ProSPC (Milipore, AB3786), anti-SMA pre-conj Cy3" (Sigma, C6198), anti-CD68 (Abcam, ab125212), anti-T1α (University of Iowa Developmental Studies Hybridoma Bank #8.1.1), anti-CD45 PE-Conj (Invitrogen, 12-0451-82). A) In naïve lungs, IDO1 is expressed in alveolar macrophages (CD68+). After BMT + γHV68, IDO1 expression increases. Macrophages (CD68+) and other cells express IDO1. B) In naïve lungs, IDO1 is expressed in vascular smooth muscle cells (αSMA+) but not in alveolar type 2 epithelial cells (proSPC +). After BMT + γHV68, IDO1 expression increases. Alveolar type 2 epithelial cells (proSPC+) do not express IDO1. Most myofibroblasts (αSMA+) do not express IDO1 (arrowheads) although some do (arrow). C) In naïve lungs, IDO1 is not expressed in alveolar type 1 epithelial cells (T1α+). After BMT + γHV68, IDO1 expression increases but IDO1 is not expressed in alveolar type 1 epithelial cells. D) In naïve lungs, IDO1 is expressed in the vascular (closed arrowheads) and airway (open arrowheads) smooth muscle and in the airway epithelium (arrows). After BMT + γHV68, IDO1 expression increases in the airway smooth muscle (open arrowheads) and airway epithelium (arrows). E) In naïve lungs, IDO1 is expressed in alveolar macrophages (CD45+, see also A)). After BMT + γHV68, IDO1 is expressed in leukocytes (CD45+), some but not all are macrophages (see A).

Figure S5

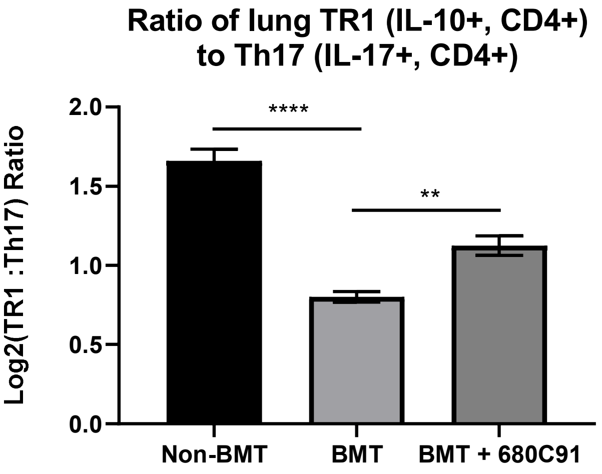

Figure S5. Inhibition of TDO2 with 680C91 restores TR1 – Th17 ratios in γHV-68 infected BMT mice. Mice were infected with γHV-68 and treated with the TDO2 inhibitor 680C91 every two days until time of harvest at 7 dpi. Lungs were harvested from groups of mice and leukocytes were purified by collagenase digestion. Leukocytes were stimulated with PMA and Ionomycin and intra-cellular cytokine staining was performed. The ratios of Tr1 to Th17 cells was calculated by dividing the Log_2(absolute cell number) of CD4+, Il-10+, IL-17- lymphocytes (Tr1) by the Log_2(absolute cell number) of CD4+, IL-17+, IL-10- lymphocytes.

Figure S6

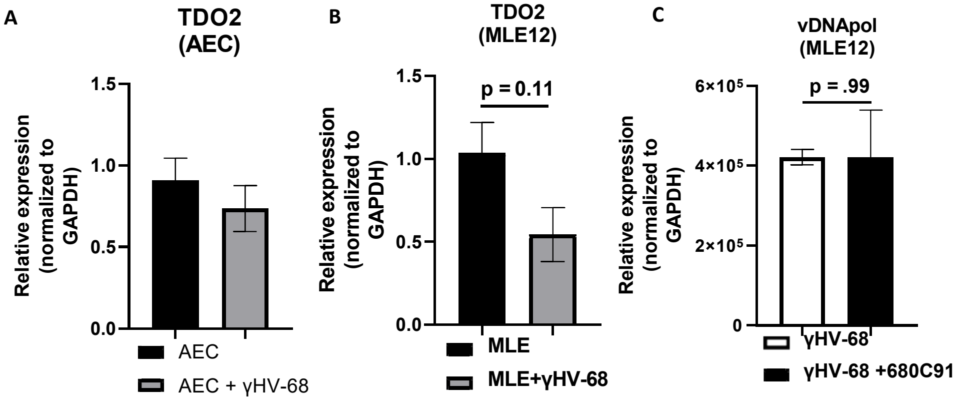

Figure S6. Alveolar epithelial cells infected with γHV-68 do not up-regulate TDO2. (A) Isolated primary alveolar epithelial cells (AEC) were infected ex-vivo at an MOI of 1.0 for 24 h after which expression of TDO2 was assessed via qRT-PCR. (B and C) Mouse lung epithelial cells (MLE12) were infected at an MOI 1.0 for 24 h after which expression of the indicated transcript was measured via qRT-PCR. Statistics were calculated using student's t-test, n = 3 samples per group.
